# Genetic basis of functional variability in adhesion G protein-coupled receptors

**DOI:** 10.1038/s41598-019-46265-x

**Published:** 2019-07-30

**Authors:** Alexander Bernd Knierim, Juliane Röthe, Mehmet Volkan Çakir, Vera Lede, Caroline Wilde, Ines Liebscher, Doreen Thor, Torsten Schöneberg

**Affiliations:** 1 Rudolf Schönheimer Institute of Biochemistry, Molecular Biochemistry, Medical Faculty, University of Leipzig, 04103 Leipzig, Germany; 2 Leipzig University Medical Center, IFB Adiposity Diseases, 04103 Leipzig, Germany

**Keywords:** Cellular signalling networks, Receptor pharmacology

## Abstract

The enormous sizes of adhesion G protein-coupled receptors (aGPCRs) go along with complex genomic exon-intron architectures giving rise to multiple mRNA variants. There is a need for a comprehensive catalog of aGPCR variants for proper evaluation of the complex functions of aGPCRs found in structural, *in vitro* and animal model studies. We used an established bioinformatics pipeline to extract, quantify and visualize mRNA variants of aGPCRs from deeply sequenced transcriptomes. Data analysis showed that aGPCRs have multiple transcription start sites even within introns and that tissue-specific splicing is frequent. On average, 19 significantly expressed transcript variants are derived from a given aGPCR gene. The domain architecture of the N terminus encoded by transcript variants often differs and N termini without or with an incomplete seven-helix transmembrane anchor as well as separate seven-helix transmembrane domains are frequently derived from aGPCR genes. Experimental analyses of selected aGPCR transcript variants revealed marked functional differences. Our analysis has an impact on a rational design of aGPCR constructs for structural analyses and gene-deficient mouse lines and provides new support for independent functions of both, the large N terminus and the transmembrane domain of aGPCRs.

## Introduction

Adhesion G protein-coupled receptors (aGPCRs) belong to an inadequately characterized class of GPCRs as their enormous size limited functional investigations for a long time^1–3^. In the last decade, however, G-protein coupling^4^, the activation mechanism by a tethered agonist^5,6^, and function as sensor for mechanical forces were identified for some members of this class^7–9^. At the physiological level, aGPCRs are involved in numerous developmental^10–13^, neural^8,14–17^, cardiovascular^11,18–21^, immune^22–26^, and endocrine processes^27–30^. Further, dysfunctions of aGPCRs are associated with human phenotypes^31^, inherited diseases^32–36^, and tumors^37–42^.

Adhesion GPCRs are nominally the second largest class of GPCRs^43,44^. Yet, reflecting the merely 33 genes encoding human representatives, this number falls behind the 719 rhodopsin-like GPCRs, while the remaining classes such as the frizzled (11 members), taste2 (25 members), secretin-like GPCR (16 members), and glutamate-like GPCR (22 members) are equally low in number^45^. However, in contrast to the majority of rhodopsin-like GPCRs^46^ all genes of aGPCRs are composed of multiple protein-coding exons spanning large genomic regions. This fragmented genomic architecture gives rise to alternative splicing often generating multiple transcript variants from a single aGPCR gene. Genome-wide reports estimate that more than 92% of human multi-exon genes produce at least two alternatively spliced variants^47,48^. For aGPCRs, several transcript variants have been reported and/or annotated in databases^9,49–54^. Even though systematic extraction of receptor variants has just started it already doubled the number of gene products in this GPCR class^49^. Some of these aGPCR variants can significantly differ in their functions as shown for GPR114^9^, EMR2^55^, latrophilins^30,56^, and GPR56^50^.

Considering the potential of multiple transcript variants with distinct functions, aGPCRs may indeed deserve the rank of the second largest GPCR class. However, a systematic analysis and quantification of aGPCR variants is still lacking. With the advent of deep-sequencing of transcriptomes and bioinformatics tools to extract the information of mRNA variants this venture becomes now, at least in parts, feasible. Therefore, in our study we aim to *i*) extract the naturally occurring transcript variants of selected aGPCRs, *ii*) estimate their relative abundance, and *iii*) translate this into the resulting structural variability at the protein level and exemplarily show what functional impact this transcript variability has *in vitro* and *in vivo*. This is of high relevance because aGPCR transcript variants can vary in their function and a profound knowledge of the existing variants is necessary to guide the design of aGPCR-directed antibodies, constructs used for structure determination, and meaningful knock-out animal models.

By extracting qualitative and quantitative data of aGPCR transcripts from very deep-sequenced RNA (RNA-seq) data of three different mouse tissues, we found that less than half of the aGPCR exons were annotated. We show that both, multiple promoters and tissue-specific splicing are responsible for the enormous transcript variability of aGPCRs. By comparing gene products at the protein level, we grouped aGPCR variants into structurally distinct gene products. We exemplarily show the impact of this data on the interpretation of aGPCR evolution, functional *in vitro* findings and phenotypes of aGPCR-targeted mouse lines.

## Results

### *De novo* transcript assembly of aGPCR transcript variants

RNA-seq data allows for quantitative expression profiling of a given gene but, if sequenced with high coverage, it can also be used for computational reconstruction and quantification of transcript variants^57–61^. To assemble mRNAs and to quantify their abundance of aGPCR genes, we used STAR^62,63^ and StringTie^58,64^ as central tools to map reads, assemble and quantify aGPCR mRNA variants in different mouse tissues (suppl. Figure S1). This tool combination has been tested and often applied^65^ because of its high performance and speed. For example, we recently applied this bioinformatics pipeline on RNA-seq data from microglia^66^. Comparing *de novo* assembled transcript variants of GPR34, an orphan rhodopsin-like GPCR, with data from PCR-based 5′ and 3′-RACE studies^67^ we found very high equivalence between the two methods^68^.

For our analysis of aGPCR transcript variants we used mouse RNA-seq datasets since genetic mouse models are the most common tool to study the functional relevance of aGPCRs and their domains. RNA-seq datasets of three different tissues (suppl. Table S1) which fulfilled our primary inclusion criteria (wild-type, biological replicates n ≥ 3, more than 100 million reads per sample, homogenous coverage of gene loci, paired-end reads) were analyzed. We found 18 different aGPCRs significantly expressed (fragments per kilobase million (FPKM) ≥0.5, suppl. Figure S2) in at least one of the three tissues (suppl. Table S2).

**Figure 1:**
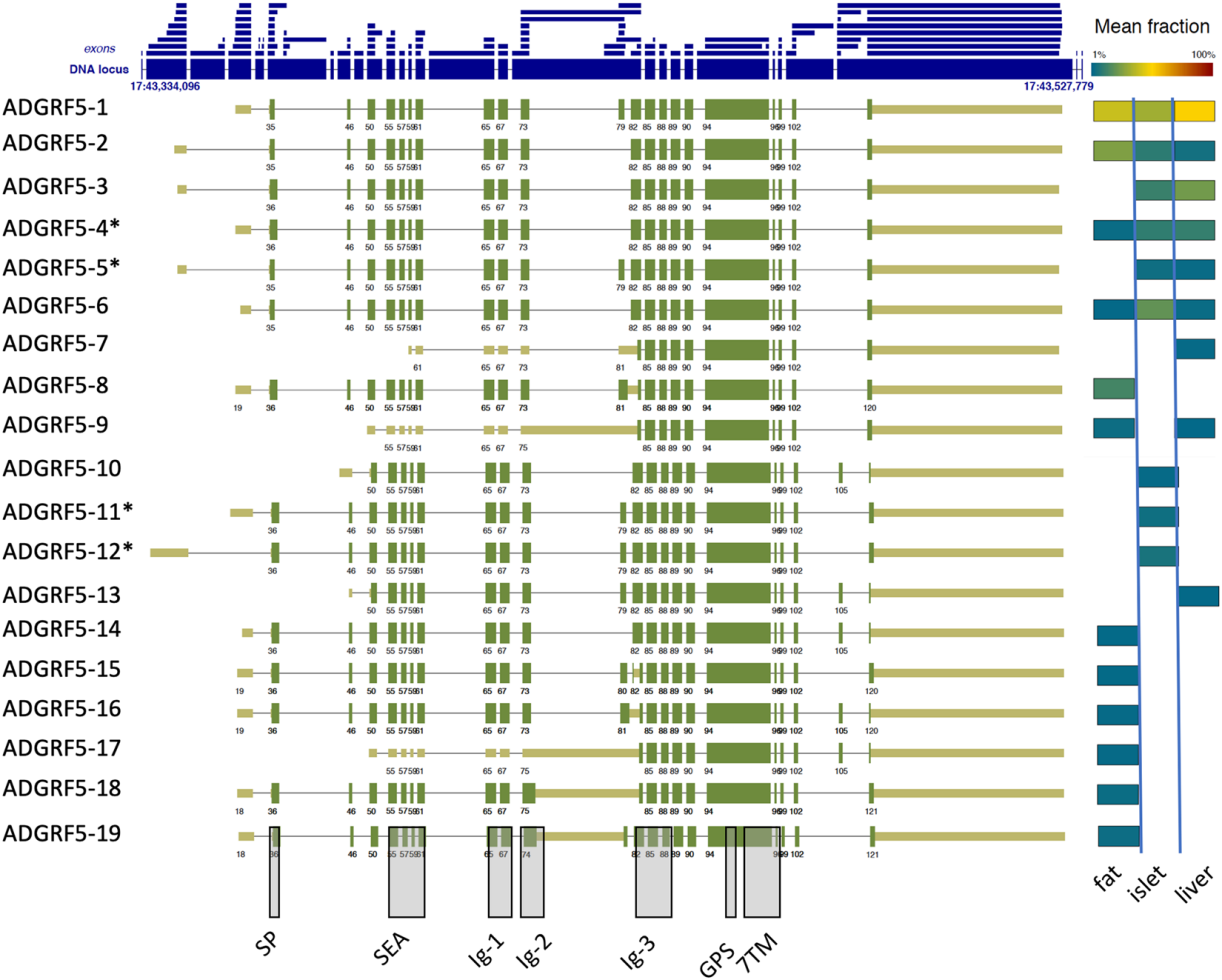
Output and visualization of ADGRF5/GPR116 transcript variants. The genomic locus of Adgrf5/Gpr116 is shown with its longest exons (large blue boxes) and size-condensed introns (faint blue lines). All exons found in the analysis are separately plotted above the locus (small blue boxes). The individual exon arrangements of transcripts are shown and numbered (e.g. ADGRF5-1). Transcripts were defined as a numeric sequence of exons (e.g. ADGRF5-1: exons 35, 46, 50 …). The longest bona fide open reading frames (ORF) are depicted in thick green boxes while the non-protein coding 5′ and 3′ UTRs are displayed thinner and in light green. 5′ start exons with minor differences in the transcription start site (TSS) but identical 3′ splice donor sites are considered as one 5′ start exon. Significantly different TSS (e.g. variant ADGRF5-3 *vs* variant ADGRF5-4) may indicate different promoters. Similarly, 3′ end exons with minor differences in length but identical 5′ splice acceptor sites are considered as one 3′ end exon. Different composition of the 5′ start exon, 3′ end exon and/or exons are considered as individual variants. The abundance of each transcript is color-coded according to the legend above. For example, variants ADGRF5-1 and ADGRF5-2 are abundant in fat tissue whereas the variant ADGRF5-5 is below 1% of all Adgrf5/Gpr116 transcripts in fat tissue or does not exist. The exact positions of the exons forming the variants are given in suppl. Table S3 and can also be visualized with genome browsers (e.g. https://software.broadinstitute.org/software/igv/download) using the provided file (Knierim et al. Suppl browser.bed). Exons, already annotated in NCBI are given in suppl. Table S3). *The variants ADGRF5-4 (XM_006524127.3, XM_006524129.2), ADGRF5-5 (XM_006524128.3), ADGRF5-11 (XM_006524124.3), and ADGRF5-12 (XM_006524125.3) show identical exon combinations as previously annotated. The grey columns indicate regions where protein domains (signal peptide (SP), Sperm protein, Enterokinase and Agrin domain (SEA), Immunoglobulin-like domain (Ig), G protein-receptor Proteolytic Site (GPS), seven-Transmembrane Domain (7TM)) are encoded.

Adgrf5/Gpr116 is one of the highly expressed aGPCRs in all three tissues (FPKM ± SD: visceral adipose tissue (VAT): 50.8 ± 9.2, liver: 7.4 ± 0.9, islets: 4.9 ± 0.1) (suppl. Table S2). Exemplarily, we use this aGPCR gene for further illustration of the bioinformatics pipeline and to guide through the analysis and results. Using all three datasets of ≥100 million reads/sample we extracted 105 different transcripts of Adgrf5/Gpr116 encoded by 79 exons (Table 1, suppl. Table S3). To visualize the results of StringTie we developed a tool which condensed the introns, color-coded the abundance of the predicted splice variants and displayed the longest ORFs together with the main structural elements. As an example, the graphical output of abundant Adgrf5/Gpr116 transcript variants (≥1% of all transcripts in the respective tissue) is given in Fig. 1. The exons map to a 200-kbp genomic region of chromosome 17 but cover only approximately 4.5% of this locus. When translated into a full-length protein the mouse ADGRF5/GPR116 has a molecular weight of up to 155 kDa. It contains several sequence signatures and domains in the extracellular N terminus such as a signal peptide (SP), a sperm protein, enterokinase, and agrin (SEA) domain, up to three immunoglobulin-like (Ig) domains and a GPCR autoproteolysis-inducing (GAIN) domain with a G protein-receptor proteolytic site (GPS). The seven transmembrane helices (7TM) domain anchors the large N terminus in the plasma membrane.

**Table 1:** Newly identified aGPCR transcript variants.

aGPCR	Old name	# annotated splice variants in NCBI	# of 5′ start exons (# of already annotated in NCBI)	# of 3′ end exons (# of already annotated in NCBI)	# of exons (# of already annotated in NCBI)	# of all variants (# of variants ≥1% abundance)	# of all exons in identified variants (# of all exon in variants ≥1% abundance)	Average # of all exons in individual variants with ≥1% abundance (min.-max. range)
ADGRL1	Lphn1	14	22 (6)	19 (1)	91 (24)	118 (56)	132 (83)	14.5 (2–25)
ADGRL2	Lphn2	51	29 (4)	24 (6)	51 (35)	108 (37)	104 (57)	15.5 (2–22)
ADGRL3	Lphn3	36	28 (3)	29 (7)	42 (36)	69 (9)	99 (37)	17.2 (2–26)
ADGRL4	Eltd1	1	16 (1)	13 (1)	22 (14)	59 (3)	51 (16)	15.4 (15–16)
ADGRE1	Emr1	4	19 (1)	9 (2)	39 (22)	52 (9)	67 (31)	17.2 (3–22)
ADGRE4	Emr4	1	16 (1)	11 (1)	31 (17)	41 (4)	58 (24)	14.3 (8–17)
ADGRE5	Cd97	7	16 (1)	13 (1)	48 (20)	117 (19)	77 (31)	17.3 (2–21)
ADGRA2	Gpr124	5	16 (2)	16 (3)	41 (19)	74 (29)	73 (49)	12.8 (3–20)
ADGRA3	Gpr125	1	17 (1)	16 (1)	31 (18)	66 (6)	64 (22)	13.2 (2–19)
ADGRC1	Celsr1	5	16 (2)	8 (1)	46 (34)	26 (9)	70 (49)	22.9 (4–35)
ADGRC2	Celsr2	5	23 (2)	15 (1)	56 (33)	49 (22)	94 (65)	20.8 (3–34)
ADGRD1	Gpr133	3	9 (2)	10 (1)	32 (26)	33 (7)	51 (31)	21.0 (10–26)
ADGRF5	Gpr116	8	23 (2)	13 (1)	43 (29)	105 (19)	79 (32)	20.8 (15–22)
ADGRB3	Bai3	4	18 (3)	6 (1)	39 (33)	41 (11)	63 (39)	18.6 (3–31)
ADGRG1	Gpr56	19	19 (10)	9 (2)	23 (15)	67 (9)	51 (21)	13.7 (5–14)
ADGRG2	Gpr64	22	17 (3)	10 (1)	35 (30)	56 (32)	62 (45)	21.8 (3–29)
ADGRG3	Gpr97	5	11 (3)	12 (1)	22 (14)	52 (24)	45 (36)	7.9 (3–12)
ADGRG6	Gpr126	7	12 (3)	6 (2)	32 (24)	29 (22)	50 (42)	16.4 (2–26)

All aGPCRs which were expressed with FPKM ≥0.5 at least in one of the three tissues were analyzed in respect to transcript variants. Following the catalog of Halvardson *et al*.^115^ newly identified exons are counted. # annotated 5′ start exons: identical splice donor; # annotated 3′ end exons: identical splice acceptor; # annotated exons: defined by a donor site and an acceptor site. A detailed analysis of the variant number and exon composition is given in suppl. Table S4.

Significant contribution to the variability of Adgrf5/Gpr116 transcripts comes from 23 different 5′ start exons (Table 1) indicating multiple transcription start sites (TSS). Thus, 5′ start exons with different 3′ splice donor sites were considered as significantly different TSS often indicating different promoters. 5′ start exons with minor differences, e.g. different transcription start points but identical 3′ splice donor sites were considered as one 5′ start exon. Similarly, 3′ end exons with minor differences in length but identical 5′ splice acceptor sites are considered as one 3′ end exon. For simplicity, we condensed the Adgrf5/Gpr116 transcript repertoire to 19 variants in Fig. 1 applying two criteria: *i*) differences in the protein-coding region and/or different 5′ start or 3′ end exons, and *ii*) an abundance of ≥1%. The abundant Adgrf5/Gpr116 variants are encoded by 32 (40.5%) out of all 79 exons of the gene. On average 20.8 exons (min. 15–max. 22 exons) built an Adgrf5/Gpr116 transcript (Table 1). Eleven exons (13.9%; mainly encoding the Ig domains, GPS, and 7TM) are included in all abundant variants. The detailed report of all identified exons, their exact position in the mouse genome, the already annotated exons, the exon composition of all assembled transcripts, the open reading frames (ORF) and the abundance of the transcripts sorted by tissues is given for Adgrf5/Gpr116 and all other aGPCRs in suppl. Table S3.

We further asked whether the read number is critical for the number of transcript variants identified in the pipeline and found a V-shaped dependency (suppl. information, suppl. Figure S3) supporting the requirement of very deep sequenced libraries. Considering this issue and using only the described parameters for including an RNA dataset (see above) there was no correlation between the FPKM of given aGPCR transcripts and the number of variants (suppl. Figure S4).

In the NCBI database there are 8 annotated Adgrf5/Gpr116 transcripts all *in silico*-assembled from shorter ESTs, RNA-seq data etc. (accession numbers given in suppl. Table S3). These annotated transcripts are derived from 32 exons (including 5′ start and 3′ end exons with major differences), which were all found in our analysis. Therefore, we now add 47 new exons that account for all transcript variants identified in the three analyzed tissues (Table 1). The exon composition of 5 annotated transcripts was identical to Adgrf5/Gpr116 transcripts we found expressed with an abundance of ≥1%. The other 3 annotated transcripts were among the low frequency transcripts or the exon combination was not found in any of our analyses (suppl. Table S3).

Single-molecule real-time (SMRT) sequencing technology (Pacific Biosciences (PacBio)) allows for analysis of complete RNA molecules without amplification. This method provides full-length transcripts without assembly and access to the direct detection of alternative splicing^69^. We analyzed a high-quality dataset (SRP101446, BioProject PRJNA374568) from neural progenitor cells and oligodendrocyte precursor cells to compare the single-molecule exon assembly of expressed aGPCRs with the predicted one from the Illumina read data. We found 30 single transcripts of 8 different aGPCRs (Adgra3/Gpr125, Adgrb3/Bai3, Adgre1/Emr1, Adgre5/Cd97, Adgrg1/Gpr56, Adgrg6/Gpr126, Adgrl2/Lphn2, Adgrl3/Lphn3) in the whole PacBio dataset of which 15 single transcripts where identical to those we predicted by our pipeline (details given in suppl. Table S3). Most of the transcripts where abundant full-length variants of those 8 aGPCRs. The remaining 15 transcripts were all shorter or truncated with skipped exons or premature breakups, respectively, but in all cases no new exons were detected. These results indicate an excellent performance of the pipeline in detection of exons and exon assembly. Additionally, in contrast to the current SMRT sequencing technology, the pipeline gives well-supported quantitative data on transcript expression.

Further support of the validity of the pipeline comes from evolutionary data. Numerous Adgrf5/Gpr116 variants are evolutionarily conserved in humans and other mammals indicating their physiological significance (see suppl. Information).

**Figure 2:**
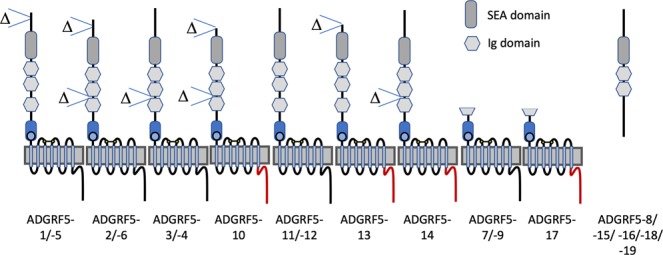
Putative (receptor) proteins resulting from Adgrf5/Gpr116 transcripts. The domain structure of proteins derived from abundant Adgrf5/Gpr116 mRNA variants (see Fig. 1) is schematically depicted. The C terminus of the receptor can also differ (red line). The exact positions of the exons forming the variants are given in suppl. Table S3.

Translation of the ORFs revealed a number of different receptor proteins. Structural variability of the translated ADGRF5/GPR116 proteins is mainly the result of alternatively spliced exons encoding the N- and the C terminus (Fig. 2). The combinations of deletions and insertions presumably shape the receptor’s N terminus of the proteins ADGRF5-1/-2/-3/-4/-5/-6. Variability of the C terminus was mainly based on frameshifting insertion and deletion of exons (e.g. ADGRF5-10/-13/-14).

As depicted in Fig. 2, several exon assemblies will cause premature truncation of the (receptor) protein resulting in potentially soluble and secreted N termini (e.g. ADGRF5-8/-15/-16/-18/-19). These transcripts still contain the ORF for downstream parts of the receptor but it remains speculative whether there is a re-initiation of translation of the mRNA^70^ leading to C-terminal receptor fragments (CTF). There are mRNA variants (ADGRF5-7/-9/-17) where the longest ORF encodes for an N-terminally truncated receptor protein consisting only of the GPS and 7TM regions. It remains speculative whether these proteins are generated and if they are correctly inserted into the endoplasmic reticulum.

In summary, the used bioinformatics pipeline is suitable to extract and assemble a comprehensive repertoire of aGPCR transcript variants. However, strict inclusion criteria (e.g. sufficient expression, saturation of the number of *de novo* assembled variants) are prerequisites for a meaningful analysis.

### Estimation of aGPCR transcript variants

Next, we used our pipeline to annotate the number and structure of transcript variants of other aGPCRs. The 18 aGPCRs, which met all inclusion criteria, showed an average of 65 variants per aGPCR gene (Table 1, suppl. Table S4). However, most transcripts differ because of sequence length diversity of the 5′ start exon and 3′ end exon. Considering only those transcripts which show an abundance of ≥1%, an average of 18 variants per aGPCR gene still remains (Table 1, suppl. Table S4). Based on this data, one can extrapolate for the 31 assigned mouse aGPCRs that more than 550 variants are significantly expressed. In average, 17 exons encode for these abundant transcripts while a regular aGPCR gene is composed of 39 exons. Considering all exons (incl. 5′ start and 3′ end exons) identified in this study, less than half (42.2 ± 11.2%) of these exons were already annotated (Table 1, suppl. Table S4). As shown in suppl. Figure S5, there is only a weak correlation between the number of exons in a given aGPCR gene and the number of derived splice variants.

In sum, the complex architecture of aGPCR genes with numerous exons and multiple promoters significantly contributes to the underappreciated repertoire of gene products.

### Structural features of proteins derived from aGPCR transcript variants

Especially the N termini of aGPCRs are structurally very diverse and composed of numerous domains, such as EGF, Ig, Pentraxin (PTX), or SEA domains. Structural variability has also been recognized within a given aGPCR protein. For example, numerical variability of defined N-terminal domains such as EGF domains in EMR2 and CD97 has been described^71,72^. Our analysis revealed that this is common in aGPCRs because 72% of all investigated aGPCRs possess splice variants changing the structure and domain composition of the N terminus (Table 2). Not only the already described numerical variation of annotated domains (Emr1, Cd97, Eltd1, Gpr116, Gpr64, Gpr126) but also the proximity between domains within the N termini (Lphn1, Lphn2, Bai3, Gpr133) can vary.

**Table 2:** Putative receptor variants derived from mouse aGPCR transcripts.

aGPCR	Old symbol	NTF domain variability	soluble NTF	membrane anchored NTF	CTF (or CTF with domain-less N terminus)	variability in 7TM	variability in C terminus
ADGRL1	Lphn1	X	X	X	X		X
ADGRL2	Lphn2	X			X		X
ADGRL3	Lphn3	X	X				X
ADGRL4	Eltd1	X					
ADGRE1	Emr1	X			X		X
ADGRE4	Emr4	X			X		X
ADGRE5	Cd97	X		X	X		
ADGRA2	Gpr124		X	X	X	X	
ADGRA3	Gpr125		X				
ADGRC1	Celsr1	X	X		X		X
ADGRC2	Celsr2		X		X		X
ADGRD1	Gpr133	X	X		X		
ADGRF5	Gpr116	X	X				X
ADGRB3	Bai3	X			X	X	
ADGRG1	Gpr56		X				
ADGRG2	Gpr64	X			X		X
ADGRG3	Gpr97		X		X		
ADGRG6	Gpr126	X		X			X
of all aGPCRs		**72.2%**	**55.6%**	**22.2%**	**66.7%**	**11.1%**	**55.6%**

Based on the ORFs of the abundant aGPCR transcript variants the resulting proteins are categorized. 7TM, seven-transmembrane domain; CTF, C-terminal fragment; NTF, N-terminal fragment.

Soluble NTFs of several aGPCRs have been identified as a result of autoproteolytic cleavage under physiological settings^73–75^. Besides proteolytic protein processing there is strong evidence that alternative splicing also contributes to the generation of soluble N termini due to frameshifts and premature stop codons. As collected in Table 2, mRNAs derived from more than half of all aGPCR genes encode for an NTF without a membrane-anchoring 7TM part. In case of Lphn1, this phenomenon can be considered as frequent. Vice versa mRNAs encoding CTF without or with a very small N terminus are also frequently found (67% of aGPCR genes). However, it remains to be tested whether such ORFs for CTFs are translated and properly inserted into the membrane despite lacking an obvious signal peptide (see below).

There are N termini which are still anchored in the membrane but lack an intact 7TM. This is found in 22% of the analyzed aGPCR genes (Cd97, Lphn1, Gpr124, Gpr126) but with low mRNA abundance. One exception is Lphn1 in VAT where membrane-anchored NTF-encoding mRNAs mount to >10% of the transcripts. There is experimental evidence that membrane-anchored N termini provide so-called *trans* signaling capacity^44^.

The 7TM domain is the most stable part with respect to alternative splicing. Only 11% of all investigated aGPCR genes show significant amounts of splice variants in this G-protein coupling mediating receptor part. Interestingly, in two cases (Bai3, Gpr124) the length of the third intracellular loop is variable because of alternative splicing. Reevaluation of public data (NCBI database) verified this finding in mouse and human BAI3 and all other members of the ADGRB group (suppl. Figure S6).

Adhesion GPCRs are not only unique because of their large N termini but also because of long C termini in some cases. C-terminal length variations (truncations) are common among aGPCRs (56%) mainly due to alternative 3′ end exons. In some cases (Lphn2, Bai2, Gpr64), there are in-frame exon insertions or deletions contributing to the variability of C termini. The C terminus of GPCRs can modulate receptor expression, trafficking, signal transduction, and interaction with an intracellular scaffold protein. Currently, there is only little information about the functional impact of the C termini of aGPCRs available. Therefore, we exemplarily tested mouse ADGRF5/GPR116 presenting variations in its C terminus (see below).

The GPS is a special structural hallmark of aGPCRs^2^. Interestingly, there are alternative splice variants in Gpr126 which lack exclusively the GPS in a TM1-anchored variant. The GPS and 7TM are often encoded by distinct exons and fused together by splicing^76^. This gives rise to functionally relevant splice variants^9^. Alternative splicing of the GPS-encoding RNA part is also found in most aGPCRs (suppl. Table S3), however, such mRNAs have a low abundance.

### Tissue-dependent differences in aGPCR variant composition

One obvious finding of our analysis was that only 15.9% and 14.1% of the 5′ start exons and 3′ end exons, respectively, were annotated in the database (Table 1, suppl. Table S4) whereas 64.3% of the classic exons were already deposited. Especially the variability in 5′ start exons can indicate multiple promoters with many of them being tissue-specific. On average, aGPCR genes have 18.2 ± 5.3 different 5′ start exons (which can contain several TSS). As an example, 17 TSS (not all at different 5′ start exons) have been previously found for human ADGRG1/GPR56^77^. In the mouse Adgrg1/Gpr56 gene, we identified 45 TSS in 16 different 5′ start exons (see dataset Adgrg1/Gpr56). The real number is probably much higher since we analyzed only 3 tissues.

Not only the promoter usage but also the pattern of transcript variants seems to be tissue-specific. Merely, one third of all aGPCRs analyzed (Gpr56, Gpr124, Gpr125, Eltd1, Emr1) shows one or two dominant forms present in all investigated tissues.

Adgrg3/Gpr97 seems to be an exception from all other aGPCRs analyzed. Although the FPKM in VAT (3.9) is comparable to liver (4.4) and significantly higher than in islets (0.56), analysis revealed only small mRNA fragments from the VAT libraries. However, in liver and islets samples full-length variants were extracted. Nevertheless, Adgrg3/Gpr97 mRNA appears more fragmented compared to other aGPCRs.

**Figure 3:**
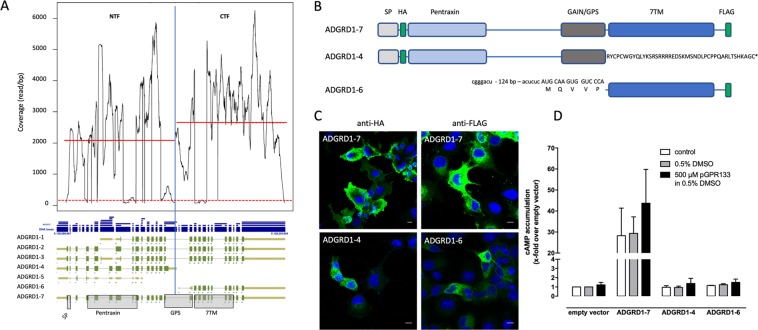
Unequal distribution of read coverage at the GPR133 locus. (**A**) Analysis of Adgrd1/Gpr133 revealed seven main transcript variants in VAT. Interestingly, two transcripts driven from different promoters encode only for the NTF (ADGRD1-4) or for the CTF (ADGRD1-6). Read coverage analysis of the NTF- and CTF-encoding genomic locus (separated by a blue vertical line) included only positions where the coverage was >1% percentile (dotted red line) to exclude bias by rare exons. The coverage per bp of the CTF-encoding exons was significantly higher (1.3 fold, p < 0.0001) as of the NTF indicating a partially dissociated transcription of both segments. The red lines mark the mean coverage in the NTF- and CTF-encoding regions. (**B**) The variants ADGRD1-4, -6, -7 were generated and N- and/or C-terminally epitope-tagged with HA and FLAG tags, respectively, as indicated. In ADGRD1-4, exon 40 (A) is used leading to a frameshift with a premature stop. The resulting amino acid sequence which is different to ADGRD1-7 is given. In ADGRD1-6, an internal promoter drives transcription starting with the GAIN coding sequence. The first AUG of the mRNA determines an ORF starting within the *Stachel* sequence 7 amino acid positions downstream the GPS. (**C**) Constructs were transiently transfected into COS-7 cells and protein expression was visualized using a monoclonal anti-HA FITC-labeled antibody (N-terminal HA tag) or a monoclonal anti-FLAG antibody/polyclonal anti-mouse FITC-labeled antibody combination (C-terminal FLAG tag). Nuclei were stained with Hoechst 33342. Pictures were taken with a confocal microscope (Zeiss, LSM 700). Bars represent 10 µm. (**D**) Constructs were transiently transfected into COS-7 cells and cAMP levels were determined in the absence/presence of an ADGRD1/GPR133-activating peptide (pGPR133)^5^ dissolved in 0.5% DMSO. Basal cAMP of vector control (pcDps) was 4.3 ± 1.3 nM. Data are given as means ± S.E.M. of three independent assays performed in triplicate. SP, signal peptide; NTF, N-terminal fragment; GAIN domain, GPCR autoproteolysis-inducing domain; GPS, G-protein coupled receptor proteolytic site; 7TM, seven-transmembrane domain; CTF, C-terminal fragment; TM transmembrane helix.

As already evident from our initial analysis (see above: inclusion criteria) there are tissue-dependent differences of the exon read coverage in some cases. Interestingly, there is also evidence that alternative promoters may even split one aGPCR gene into two separate genes. For example, there are Adgrd1/Gpr133 transcripts in VAT encoding the NTF and the CTF separately using two different promoters. Coverage analysis revealed an asymmetric abundance of the NTF- and CTF-encoding fragments (Fig. 3A). A similar separation of the NTF and CTF is seen for Lphn1 and Celsr2. More frequently, there are promoters separating the CTF from the NTF as an individual gene as observed for Bai3, Gpr97, Emr1, Emr4, and Lphn2 again producing a higher coverage of the CTF encoding gene portion (see suppl. Table S3). Using Adgrgd1/Gpr133 as example, we analyzed whether transcriptionally generated NTF and CTF are indeed produced as proteins. We cloned the full-length (ADGRD1-7), NTF (ADGRD1-4) and CTF (ADGRD1-6) transcript variants (Fig. 3B) into the mammalian expression vector pcDps and expressed them transiently in COS-7 cells. As shown in Fig. 3C, all constructs were found to be expressed as proteins by immunofluorescence studies, however, the CTF (ADGRD1-6) construct to a lesser extent in the endoplasmic reticulum (ER). As expected for the signal peptide-containing NTF (ADGRD1-4), the protein was found in the ER. The full-length construct (ADGRD1-7) showed increased basal activity compared to vector control as reported before^4^ and can be stimulated with a *Stachel*-sequence derived peptide (Fig. 3D)^5^. The NTF (ADGRD1-4) and CTF (ADGRD1-6) did not show increased basal activity most probably because of the lack of the 7TM and an N-terminally truncated *Stachel* sequence, respectively.

In sum, transcript heterogeneity of aGPCRs is caused by alternative promoter usage and splicing. One can, therefore, speculate that the number of exons and variants will further increase with the number of investigated tissues and cell types. There is now experimental evidence that even transcripts encoding for partial aGPCR variants are translated into proteins.

### Impact of transcript variants on aGPCR phylogeny, function and transgenic mouse models

The knowledge of the transcript repertoire has substantial implications on e.g. phylogenetic considerations, functional testing of variants, and the design of transgenic mouse models. Exemplarily, we tested the relevance of data on the transcript repertoire with respect to *i)* evolutionary relations of aGPCRs, *ii)* impact on ADGRF5/GPR116 function, and *iii)* mouse models for Adgrf5/Gpr116 deficiency in the following subsections.

### Exon-intron architecture and phylogenetic relations

**Figure 4:**
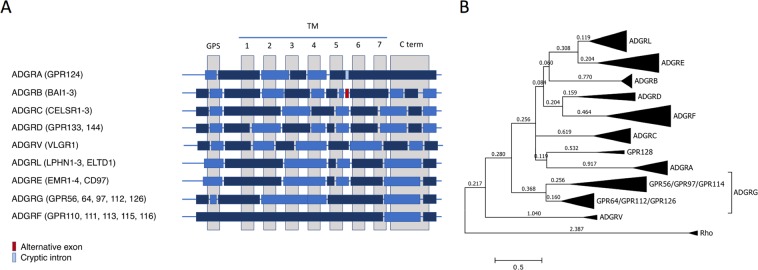
Exon-intron architecture of the 7TM-encoding genomic region of aGPCR and its implication in aGPCR phylogeny. **(A)** Based on our mRNA variant analysis and publicly available genomic data the exon-intron structure of aGPCR groups is schematically presented. Alternating dark and light blue boxes represent GPS- and 7TM-encoding exons which are interrupted by introns. **(B)** The evolutionary history of vertebrate aGPCRs (human, mouse, chicken, zebrafish orthologs) was inferred by using the Maximum Likelihood method based on the JTT matrix-based model^116^. Thus, the 7TM domain of human, mouse, chicken, and zebrafish aGPCR orthologs were aligned and the tree with the highest log likelihood (-21466.21) is shown. Rhodopsin was used as outgroup. Initial tree(s) for the heuristic search were obtained automatically by applying Neighbor-Join and BioNJ algorithms to a matrix of pairwise distances estimated using a JTT model, and then selecting the topology with superior log likelihood value. The tree is drawn to scale, with branch lengths measured in the number of substitutions per site (next to the branches). The analysis involved 133 amino acid sequences. All positions containing gaps and missing data were eliminated. There were a total of 170 positions in the final dataset. Evolutionary analyses were conducted in MEGA7^117^.

The 7TM domain is the most conserved structure and has been used to analyze the phylogenetic relation and to establish the classification of aGPCRs forming 9 major groups (Fig. 4A)^2^. Transcript analysis also revealed the genomic exon-intron architecture which can be useful to determine the phylogenetic relations between 7TM domains of GPCRs^78,79^. As shown in Fig. 4, all aGPCR genes except for the ADGRF group present a complex exon-intron-structure of the 7TM-enconding region. Obviously, ADGRL and ADGRE share the same organization of the 7TM-encoding genomic region indicating their close evolutionary relation. Phylogenetic evaluation in different models shows that ADGRL and ADGRE share branch lengths which are usually found within aGPCR groups (Fig. 4B). For example, two subgroups within the ADGRG group containing GPR56/GRP97/GPR114 and GPR64/GPR112/GPR126 show longer branch lengths than branches separating ADGRL and ADGRE members (Fig. 4B). Therefore, one may consider the aGPCRs of ADGRL and ADGRE as members of just one group.

In contrast to all other aGPCR groups, the GPS and most of the 7TM of the ADGRF group are encoded by a single exon (Fig. 4A). Because there are no direct ADGRF orthologs in invertebrates it is very likely that the ADGRF group derived from the genomic integration of a processed mRNA and reverse transcript cDNA which reintegrated into the genome and underwent gene duplications in early vertebrate evolution.

### Functional relevance of the length variability of the N- and C termini

**Figure 5:**
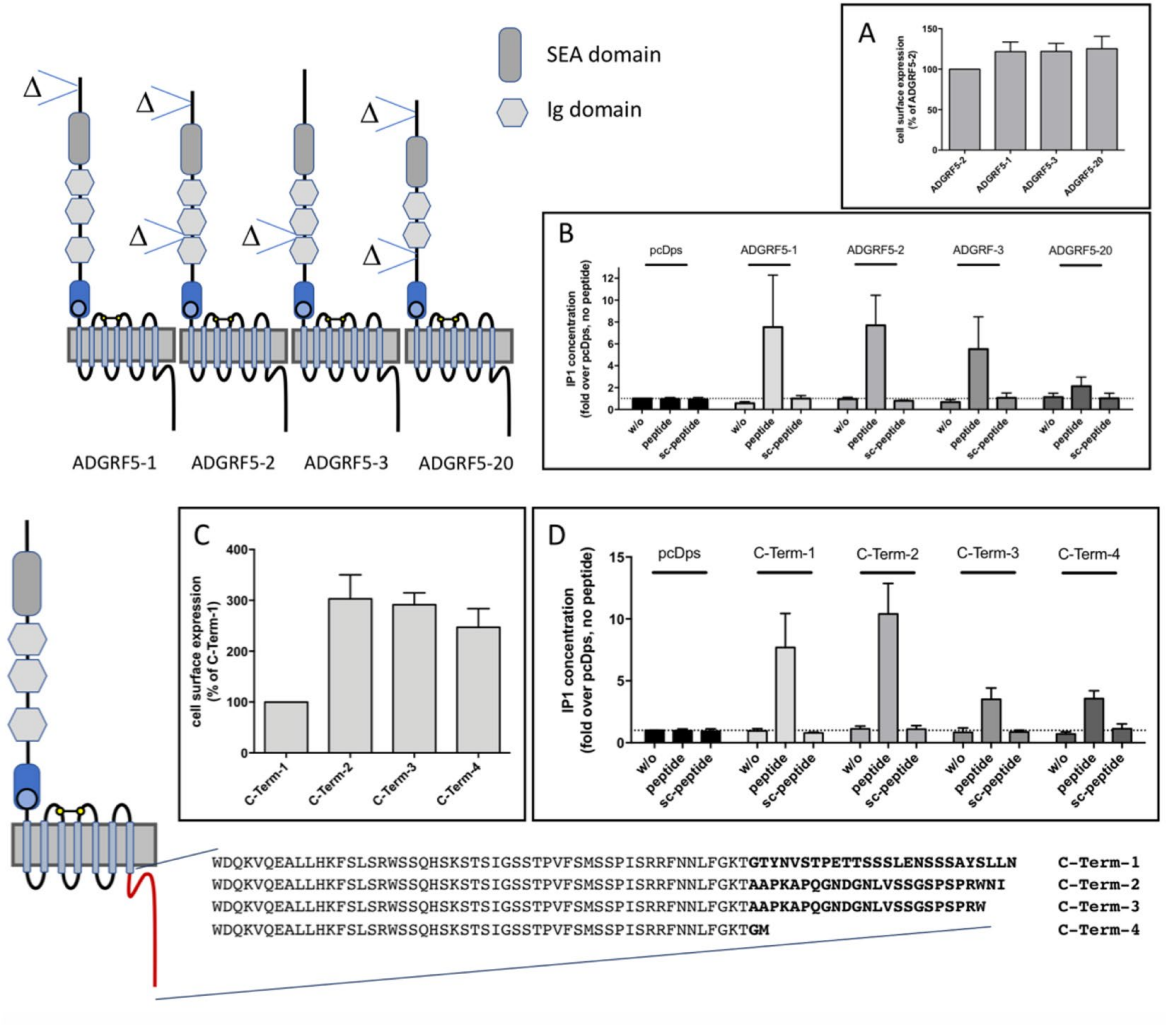
Functional impact of N- and C terminus length variations in mouse ADGRF5/GPR116. Splice variant analysis of Adgrf5/Gpr116 revealed several variations of the N- and C terminus lengths. Selected variants were tested in respect to their cell surface expression and signal transduction properties. The common N-terminal variants ADGRF5-1-3, a rare variant that lacks the third Ig domain (ADGRF5-20, suppl. Table S3 Adgrf5/Gpr116 variant fat 2_6) and the empty vector (pcDps) were tested in (**A**) cell surface expression ELISA and (**B**) inositol phosphate (IP1) assays. In IP1 assays the variants were analyzed without (w/o) and with the *Stachel* peptide of GPR116 (1 mM) or a scrambled peptide as control (1 mM). Similarly, four selected mouse GPR116 variants differing in their C-terminus lengths were tested. C-Term-1 and C-Term-2 correspond to ADGRF5-1-9, -15, -18, -19 and ADGRF5-10, -13, -14, -16, -17 of Fig. 2, respectively. C-Term-3 and C-Term-4 were rare splice variants (<1% of all GPR116 transcripts) in the data sets we analyzed. (**C**) Cell surface expression (ELISA) and (**D**) agonist-induced inositol phosphate (IP1) accumulation assays were performed. Data are given as means ± S.E.M. ELISA OD pcDps: 0.006 ± 0.003 (N-Term) and 0.008 ± 0.004 (C-Term), AGDRF5-2: 0.120 ± 0.021, C-Term-1: 0.105 ± 0.023; as positive control (not shown) the HA-tagged ADP receptor P2RY12 showed an OD value of 0.322 ± 0.041. IP1: pcDps w/o: 215 ± 37 nM, n ≥ 4 (C-Term) and n ≥ 5 (N-Term).

Over 70% and 50% of the investigated aGPCRs show length variabilities of their N- and C termini (Table 2), respectively. As an example, we tested the functional consequences of 4 N-terminal and 4 C-terminal variants of ADGRF5/GPR116 with respect to their expression and *Stachel* peptide-induced signal transduction. As shown in Fig. 5A, cell surface expression of ADGRF5/GPR116 variants did not significantly differ and agonist-induced IP1 formation corresponded to the cell surface expression of the individual N-terminal variants ADGRF5-1, -2 and -3 (Fig. 5B). In our transcript analysis, we mainly identified ADGRF5/GPR116 variants with 3 putative Ig domains but also variants containing only two as previously described^43,80,81^. Interestingly, although the deletion of the third Ig domain in ADGRF5-20 did not influence the cell surface expression of this variant (Fig. 5A), a complete loss of peptide agonist-mediated inositol phosphate formation was observed (Fig. 5B) indicating some functional impact of the N terminus on the 7TM.

As shown in Fig. 5C, the cell surface expression of the longest C-terminal variant-1 is significantly lower compared to the three other variants. However, signaling efficacy is unchanged between variant-1 and variant-2 whereas variants-3 and -4 display a reduced *Stachel*-induced IP1 formation (Fig. 5D). This indicates that the C terminus of ADGRF5/GPR116 contributes to receptor trafficking and Gq protein-mediated signaling. A recent study analyzing cancer-specific splicing in more than 1,000 patients identified a non-canonical ADGRF5/GPR116 isoform with an altered C terminus representing an alternative spliced ADGRF5/GPR116 variant (Fig. 1). Moreover, this isoform is associated with poor prognosis^82^. Based on the data provided in this study, this variant is most probably our C-terminal variant-2 (Fig. 5C/D) using exon 105 (Fig. 1).

### Impact of transcript variants on the generation of aGPCR-deficient mouse lines

Previous studies already showed different phenotypes in transgenic mouse lines although the same aGPCR gene was targeted. For example, two Adgrg6/Gpr126-targeted deletion mouse lines revealed distinct phenotypes, mid-gestation lethality with cardiovascular malformations^10^ and vital newborns with hypomyelinated peripheral nerves which died before weaning^83^. Considering that aGPCRs are composed of dozens of exons and that expression is driven by different promoters, every mouse line in which aGPCRs are targeted needs to be evaluated in respect to transcript composition and quantity.

**Figure 6:**
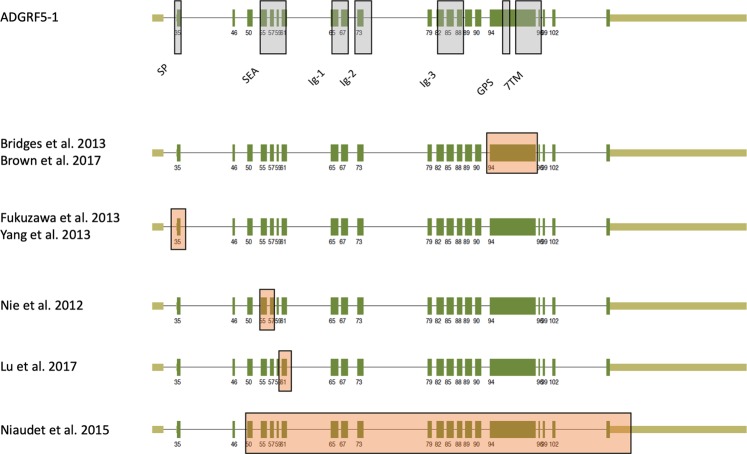
Adgrf5/Gpr116 locus targeted in mouse lines. There are several mouse lines in which the GPR116/ADGRF5 locus was targeted disrupting individual exons^19,84–89^. The exon and domain annotation of ADGRF5-1 is taken from Fig. 1. Orange boxed exons mark the deletion in the different mouse lines.

As an example, we reevaluated all available mouse lines targeting the Adgrf5/Gpr116 locus on the basis of our transcript variant data (Fig. 1). As shown in Fig. 6, exon 35 (former exon 2), exons 55 and 57 (former exons 5–6), exon 61 (former exon 8), exon 94 (former exon 17) and exons 50–121 (former exons 4–21) were deleted in the different mouse lines published^19,84–89^. Although deletion of the individual exons may produce N- or C-terminally truncated ORFs (exon deletions: 35, 94, or 50–121) or ORFs with frameshifts (exons 55 and 57, or 61), the promoters used for ADGRF5-7, -10, -13, (Fig. 1) will produce Adgrf5/Gpr116 transcripts even in the absence of these exons. Furthermore, there is a possibility of exon skipping which may produce mRNA with an ORF of the partial wt sequence. Indeed, we found abundant Adgrf5/Gpr116 transcripts from the exon 94 deletion mouse line^84^ fusing the NTF to most of the transmembrane helix 7 and anchoring the complete N terminus within the plasma membrane (unpublished results). Reflecting the fact that aGPCRs may use their NTF for *trans* signaling the different mouse lines might present partial phenotypes because of remaining receptor portions. In case of ADGRF5/GPR116 this may explain the graduate phenotypic differences between the mouse lines in respect to the onset of the dysregulated surfactant production, heart weight, and vascular function of ADGRF5/GPR116^19,84–89^.

Taken together, detailed knowledge about naturally occurring transcript variants helps to better assign the exon structure of aGPCR genes and provides not only important information for proper design of genetic animal model but also sheds light on the evolutionary relation of aGPCR members. Our functional analysis of Adgrf5/Gpr116 transcript variants exemplarily highlights the fact, that variants can mainly differ in their expression and signal transduction. It is therefore of importance to individually test all significantly expressed transcript variants to provide a comprehensive picture of their biological functions.

## Discussion

The wealth of RNA-seq data makes it nowadays feasible to generate comprehensive catalogs of transcript variations in different organisms, tissues and cell types. Many computational methods have been developed to assemble transcripts from short RNA-seq reads with some differences in their performance^90,91^. However, the combination of multiple exons in very long transcripts, as it is the case for most aGPCRs, is still challenging^92^ and the exon-exon read support and read abundance of exons are mainly utilized for transcript phasing. Therefore, detailed evaluation of the results of computational methods for transcript reconstruction and quantification from RNA-seq data is necessary. We evaluated our results by comparing transcripts of different tissues (e.g. Fig. 1), assuring saturation of transcript *de novo* assembly (suppl. Figure S3) and independence of the results from FPKM values (suppl. Figure S4) and by comparing our data with already annotated transcripts. However, one should keep in mind that long transcripts annotated from experimental data can also be “artificially assembled” because long-range PCR (e.g. RACE strategies) is prone to produce chimera from overlapping fragments. Currently, only RNA-seq data provides saturating experimental support for exon-exon junctions and quantitative data of exon abundance. Some advantage comes from improved long-fragment sequencing technologies. Here, the combination of exons in a single mRNA molecule can currently be analyzed with the single-molecule sequencing technology by Pacific Biosciences. This sequencing technology produces reads up to a few tens of thousands of base pairs. Indeed, several long aGPCR mRNAs could be extracted from public PacBio datasets verifying predicted variants (see above). However, this technology is far from providing quantitative data and raw reads display significantly higher error rates (∼10–20%) than reads from the Illumina technology (∼1%)^93^.

Being aware of all these limitations, we defined our RNA-seq dataset inclusion parameters very restrictive which were only fulfilled by 3 datasets of different mouse tissues (islet, liver, VAT). First, we evaluated our pipeline on Adgrf5/Gpr116 which shows very different expression levels and found that saturation of extracted mRNA variants requires FPKM values > 0.5 and >100 million reads per sample (suppl. Figure S3B). Further, we never missed an already annotated exon and longest ORF in Adgrf5/Gpr116 (and all the 18 other aGPCR genes). This already indicates a good performance of the applied variant annotation pipeline. However, we did not find all exon combinations annotated in full-length Adgrf5/Gpr116 isoforms. Inspection of the isoforms already annotated in NCBI revealed that the exon combination of the full-length variants was not based on experimental data but was rather an artificial product by introducing exon-exon support (e.g. from EST or RT-PCR fragment) into an already existing GPR116 variant. This is actually true for many, if not for most, aGPCR isoforms annotated in NCBI.

On average, we found 18 mRNA variants per aGPCR gene when we consider only those that are significantly expressed (suppl. Table S4). Variants that differed only in the 5′ position of the first or the 3′ position of the last exon were treated as one variant. In a recent genome-wide analysis five alternative splice variants are derived from an average human gene and there are quite a few genes with more than 10 splice variants^90^. Unfortunately, aGPCRs were not included in this analysis. However, aGPCR genes appear to be on the upper level of mRNA variants among all genes most probably because they have also a high number of exons (suppl. Figure S5).

The modular domain architecture, e.g. of EGF domains, has been previously described for some aGPCRs^71,72^. This seems to be a general phenomenon because the coding regions for the N termini differ in more than two thirds of all investigated aGPCR transcripts leading to changes in the domain architecture of the NTF (Table 2). Less frequent are variations of the 7TM and the C terminus. We exemplarily demonstrated that even small changes in the structure of the N- and C termini can have an impact on cell surface expression and G protein-mediated signal transduction (Fig. 5). The biological significance of many transcript variants is documented by the evolutionary conservation in mouse and human orthologs over 180 million years (e.g. suppl. Figure S6). Further, the existence of NTFs and CTFs was previously related to autoproteolytic cleavage at the GPS^94^ but not to transcript variants. In this study, we found evidence that CTFs and NTFs are separately generated by distinct promoters (Fig. 3). This supports the hypothesis that the separate NTF and CTF were genetically recombined producing a receptor fusion protein^95^. However, our data now provide strong evidence that in some aGPCR genes the NTF- and CTF-encoding parts still function as separate genes translated into individual proteins (Fig. 3). The physiological relevance of these only NTF- and 7TM-encoding transcripts needs to be studied in the future.

Projection of RNA-seq data on the genome provides information about the architecture of aGPCR genes. Here, we found close relation of the genomic structure of the ADGRL and ADGRE groups (Fig. 4A). Phylogenetic analysis showed that the differences at the amino acid level between these two aGPCR groups are smaller compared to differences within e.g. the ADGRG group (Fig. 4B). Since the members of ADGRG group share similar genomic exon architecture and the current aGPCR nomenclature is based on amino acid sequence similarities^2^ one must reconsider ADGRL and ADGRE being only one group.

Our data further demonstrates that a thoughtful RNA-seq-based annotation of the genomic architecture of aGPCR genes is required for proper interpretation of phenotypes found in aGPCR gene-targeted mouse lines (Fig. 6). Here, differences in phenotypes from mouse lines targeting the same aGPCR gene may result from partial deletions or artificially generated transcripts. We, therefore, suggest to perform RNA-seq as standard analysis to characterize the transcript repertoire resulting from a targeted aGPCR locus prior to and after transgenic manipulation.

Finally, aGPCRs are the remaining GPCR class where no crystal structure of a full-length protein is available yet^96^. The successful crystallization of GPCRs over the last decade is mainly based on detailed knowledge of structure-function relationships. Stabilization of the 7TM domain by interaction partner^97^, directed mutagenesis^98^, and introduction of fusion protein domains^99^ supported crystallization attempts. In great contrast to rhodopsin-like GPCRs, there is only very little of such essential information about structure-function relationships of aGPCRs available. Data from naturally occurring variants can help in rational designing of aGPCR constructs to increase expression and stability of the receptor proteins.

Our in-depth transcript characterization of mouse aGPCRs provides an unexpected broad transcript repertoire which is often tissue-specific. Multiple exon combinations and intra-gene TSS are responsible for modular domain assembly of aGPCRs and receptor fragments. This structural variability together with the ability for *cis* and *trans* signaling make aGPCRs unique among the superfamily of GPCRs. Cross-species analyses, variant-specific functional *in vitro-* and *in vivo* studies, and analyses of individual variants with crystallography or cryo-electron microscopy may help to dissect the functional relevance of this exceptional receptor repertoire.

## Methods

### RNA-seq data, workflow to extract and quantify aGPCR variants

The general workflow to extract aGPCR transcript variants from Illumina RNA-seq data is given in suppl. Figure S1. RNA-seq datasets from NCBI Sequence Read Archive (SRA)^100^ were chosen to allow analyses of multiple aGPCRs in wild-type mice with at least three biological replicates per tissue. Other inclusion criteria were paired-end reads with a length ≥100 base pairs and a high sequencing depth (≥100 million reads/sample). Datasets generated without random primers were excluded.

The sequences were aligned to the current reference mouse genome (mm10/GRCm38) using the splice aware aligner STAR (version 2.5.2)^62,63^. After indexing with samtools (version 1.3.1)^101^ the mapped reads were assembled to transcripts and quantified by StringTie (version 1.3.3)^58,64^. For STAR, we used the ‘default’ parameters which are commonly used in most studies. StringTie parameters ‘read coverage’ (-c), ‘transcript length’ (-m) and ‘bases on both sides of a junction a spliced read has to cover’ (-a) were set to minimal values in order to avoid missing transcripts and generating a bias. The parameter ‘fraction of most abundant transcript at one locus’ (-f) was lowered from default (0.01) to 0 since correction for artifacts and incompletely processed mRNA with a 1% cutoff was performed after the comparative analysis. For all other StringTie parameters default values were used.

Assembled transcripts were inspected with the Integrated Genome Viewer (Broad Institute) (version 2.3.91)^102,103^ and samples showing a visible 3′ bias due to oligo-dT/poly-A primer selection were not included. FPKM values of all aGPCRs were determined with bamUtils^104^ and libraries with more than 3 aGPCRs having a FPKM ≥0.5 were included in the analysis. This value was taken from a pre-analysis which showed that the average median of all FPKM values is 0.42 (Figure S2). For this analysis, we included the FPKM values of all transcripts in a sample and calculated the FPKM median which than was averaged for the medians of all samples. Therefore, the cut-off of 0.5 was defined as rounded value of the averaged FPKM median.

Screening of NCBI SRA revealed only three mouse datasets meeting all criteria: visceral adipose tissue (VAT), liver, and pancreatic islets (suppl. Table S1).

For comparing the transcripts across multiple samples from different tissues, all exons of every aGPCR transcript were aligned and numbered consecutively using R (version 3.4.2). Transcripts were defined as a numeric sequence of exons. The nucleotide sequence of each exon was extracted with bedtools (version 2.25.0)^105,106^ and the longest open-reading frame (ORF) of each transcript was identified and translated from the assembled full-length mRNA sequence using the *seqinr* R package (version 3.4–5)^107^. The abundance of each assembled transcript was determined with StringTie for each sample and transcripts with an average abundance of ≥1% were considered in further analyses. The resulting amino acid sequence was then screened for annotated protein domains deposited in the Uniprot database^108^.

For visualization of the quantity of the different transcripts in different tissues, a script was developed with R^109^ plotting the aGPCR locus with experimentally supported exons and condensed intron sizes.

### Analysis pipeline of PacBio data

To investigate the exon composition of long aGPCR mRNAs, the raw sequence reads of public dataset SRP101446 belonging to the BioProject PRJNA374568 were downloaded from the SRA database. This data set is a collection of single sequence reads from *Mus musculus* neural progenitor cells and oligodendrocyte precursor cells. In the dataset, there are two kinds of files coming from different sequencing platforms (Illumina NextSeq 500, PacBio RS II). Only reads that are generated by PacBio RS II platform (Pacific Biosciences) were used for the validation of splice variants in our analysis due to its read lengths of kilobases so that a single read can cover possibly a whole transcript (suppl. Figure S6).

In our pipeline first SMRTbell adapters and poly-A tails were removed by the open source tool BBMap (https://sourceforge.net/projects/bbmap/). The clipped data had average read number of 497,138 and average read length of 3,252. Then clipped reads were mapped to the reference genome (*Mus musculus* mm10 assembly, Ensembl database) by using segemehl sequencing read aligner^110,111^. Segemehl was used due to its certain advantages that fit to our purpose; *i*) no limitation to a specific read length, *ii*) option to use split read alignment and *iii*) high sensitivity^112^. For analysis of PacBio data, the segemehl parameters were default values with split read functionality. After mapping, reads that mapped to genes of interest were collected. As expected, transcripts, that had reads mapped to a gene, were mostly covered by splits of single reads, which rendered us to directly identify splice variants. Of note, apart from the fact that PacBio read length enables one to cover a bigger region at once, because of the low coverage this method is not suitable to quantify transcripts yet, but provides a qualitative validation.

### Generation of aGPCR variants, expression, and second messenger assay

Mouse ADGRF5/GPR116 constructs for functional analyses were generated by cloning cDNA from total mRNA of mouse heart^113^ and mouse lung (splice variants) into the mammalian expression vector pcDps. RNA was isolated using the ReliaPrep RNA Cell Miniprep System (Promega). cDNA was obtained with a reverse transcriptase (RT) and oligo-dT primers. The full-length mouse Adgrd1/Gpr133 was from the previously reported study^4^. Variants were generated by PCR fragment replacement strategies. All constructs were epitope-tagged with an N-terminal hemagglutinin (HA) tag (YPYDVPDYA) and/or a C-terminal FLAG tag (DYKDDDDK). The coding sequence of the HA tag was inserted directly 3′ of the signal peptide-encoding sequence of the variants. The coding sequence of the FLAG tag was inserted 5′ of the natural stop codon. The correctness of the constructs was verified by Sanger sequencing.

For heterologous functional assays, COS-7 cells were transiently transfected. COS-7 cells were grown in Dulbecco’s modified Eagle medium (DMEM) supplemented with 10% fetal bovine serum, 100 U/ml penicillin, and 100 μg/ml streptomycin at 37 °C in a humidified incubator with 5% CO_2_. To determine the cell surface expression of receptors carrying an N-terminal HA tag, a cellular enzyme-linked immunosorbent assay (ELISA) was used. Thus, COS-7 cells were split into 48-well plates (6 × 10^4^ cells/well) for cell surface expression ELISA and into 96-well plates (3.5 × 10^4^ cells/well) for inositol phosphate (IP1) assay. Transient transfection (4 µg receptor-encoding plasmid DNA/T25 culture flask) was performed using Lipofectamine2000 (Thermo Fisher Scientific) according to manufacturer’s protocol and split into the multi-well plates 24 hours post transfection. For determination of cell surface expression, receptors were analyzed with anti-HA-peroxidase (Roche) in cellular ELISA as described previously^114^.

IP1 and cAMP formations were induced by incubation with 1 mM peptides for 30 minutes and determined with the IP-One Tb kit (Cisbio) and the Alpha Screen cAMP assay kit (PerkinElmer Life Sciences), respectively,  as previously described^113^.

For imaging ADGRD1 variants, COS-7 cells were transfected with the indicated constructs. The ADGRD1 variants were cloned from PCR fragments using mRNA from leucocytes and primers designed to amplify the desired variants (see Fig. 3B). The cDNAs encoding the ADGRD1 variants were cloned into the mammalian expression vector pcDps. The variants were epitope-tagged with an N-terminal hemagglutinin (HA) tag and/or a C-terminal FLAG tag as indicated in Fig. 3B. The coding sequences of the HA and FLAG tags were inserted directly 3′ of the signal peptide-encoding sequence and 5′ of the natural stop codon of the variants, respectively. 48 h after transfection, COS-7 cells previously seeded on cover slips into 12-well plates (15 × 10^4^ cells/well), were fixed, and mounted on glass slides. Protein expression was visualized using a monoclonal anti-HA antibody (N-terminal HA tag, Sigma-Aldrich, H3663) or a monoclonal anti-FLAG antibody (C-terminal FLAG tag, Sigma-Aldrich, F1804) with polyclonal anti mouse FITC-labeled antibody (Sigma-Aldrich, F9137) combination. Nuclei were stained with Hoechst 33342 (Sigma-Aldrich). Images were taken with a confocal laser-scanning microscope (LSM 700; Carl Zeiss Jena Gmbh, Jena, Germany).

Assay data was analyzed with GraphPad Prism version 7.0. Statistics were performed using a one-way ANOVA with a Bonferroni post-hoc test or unpaired student’s t-test.

## Supplementary information

Supplementary

Table S1

Table S2

Table S3

Table S4

Gene Browser data

## Data Availability

All RNA-Seq data are either available from public resources or given in the supplementary material.

## References

[1] Liebscher I, Schoneberg T, Promel S (2013). Progress in demystification of adhesion G protein-coupled receptors. Biol Chem.

[2] Hamann J (2015). International Union of Basic and Clinical Pharmacology. XCIV. Adhesion G protein-coupled receptors. Pharmacol Rev.

[3] Liebscher I, Monk KR, Schoneberg T (2015). How to wake a giant. Oncotarget.

[4] Bohnekamp J, Schoneberg T (2011). Cell adhesion receptor GPR133 couples to Gs protein. J Biol Chem.

[5] Liebscher I (2014). A tethered agonist within the ectodomain activates the adhesion G protein-coupled receptors GPR126 and GPR133. Cell Rep.

[6] Stoveken HM, Hajduczok AG, Xu L, Tall GG (2015). Adhesion G protein-coupled receptors are activated by exposure of a cryptic tethered agonist. Proc Natl Acad Sci USA.

[7] Scholz N (2015). The adhesion GPCR latrophilin/CIRL shapes mechanosensation. Cell Rep.

[8] Petersen SC (2015). The adhesion GPCR GPR126 has distinct, domain-dependent functions in Schwann cell development mediated by interaction with laminin-211. Neuron.

[9] Wilde C (2016). The constitutive activity of the adhesion GPCR GPR114/ADGRG5 is mediated by its tethered agonist. FASEB J.

[10] Waller-Evans H (2010). The orphan adhesion-GPCR GPR126 is required for embryonic development in the mouse. PLoS One.

[11] Kuhnert F (2010). Essential regulation of CNS angiogenesis by the orphan G protein-coupled receptor GPR124. Science.

[12] Langenhan T (2009). Latrophilin signaling links anterior-posterior tissue polarity and oriented cell divisions in the C. elegans embryo. Dev Cell.

[13] Koirala S, Jin Z, Piao X, Corfas G (2009). GPR56-regulated granule cell adhesion is essential for rostral cerebellar development. J Neurosci.

[14] Tu YK, Duman JG, Tolias KF (2018). The Adhesion-GPCR BAI1 Promotes Excitatory Synaptogenesis by Coordinating Bidirectional Trans-synaptic Signaling. J Neurosci.

[15] Duman JG (2013). The adhesion-GPCR BAI1 regulates synaptogenesis by controlling the recruitment of the Par3/Tiam1 polarity complex to synaptic sites. J Neurosci.

[16] Anderson GR (2017). Postsynaptic adhesion GPCR latrophilin-2 mediates target recognition in entorhinal-hippocampal synapse assembly. J Cell Biol.

[17] O’Sullivan ML (2012). FLRT proteins are endogenous latrophilin ligands and regulate excitatory synapse development. Neuron.

[18] Patra C (2013). Organ-specific function of adhesion G protein-coupled receptor GPR126 is domain-dependent. Proc Natl Acad Sci USA.

[19] Lu S (2017). Developmental vascular remodeling defects and postnatal kidney failure in mice lacking Gpr116 (Adgrf5) and Eltd1 (Adgrl4). PLoS One.

[20] Masiero M (2013). A core human primary tumor angiogenesis signature identifies the endothelial orphan receptor ELTD1 as a key regulator of angiogenesis. Cancer Cell.

[21] Xiao J (2012). Augmented cardiac hypertrophy in response to pressure overload in mice lacking ELTD1. PLoS One.

[22] Das S (2011). Brain angiogenesis inhibitor 1 (BAI1) is a pattern recognition receptor that mediates macrophage binding and engulfment of Gram-negative bacteria. Proc Natl Acad Sci USA.

[23] Park D (2007). BAI1 is an engulfment receptor for apoptotic cells upstream of the ELMO/Dock180/Rac module. Nature.

[24] Lin HH (2017). Adhesion GPCRs in Regulating Immune Responses and Inflammation. Adv Immunol.

[25] Hamann J, Hsiao CC, Lee CS, Ravichandran KS, Lin HH (2016). Adhesion GPCRs as Modulators of Immune Cell Function. Handb Exp Pharmacol.

[26] Wang JJ (2013). Gpr97 is essential for the follicular versus marginal zone B-lymphocyte fate decision. Cell Death Dis.

[27] Duner P (2016). Adhesion G Protein-Coupled Receptor G1 (ADGRG1/GPR56) and Pancreatic beta-Cell Function. J Clin Endocrinol Metab.

[28] Gupta R (2018). Complement 1q-like-3 protein inhibits insulin secretion from pancreatic beta-cells via the cell adhesion G protein-coupled receptor BAI3. J Biol Chem.

[29] Balenga N (2017). Orphan Adhesion GPCR GPR64/ADGRG2 Is Overexpressed in Parathyroid Tumors and Attenuates Calcium-Sensing Receptor-Mediated Signaling. J Bone Miner Res.

[30] Rothe J (2019). Involvement of the Adhesion GPCRs Latrophilins in the Regulation of Insulin Release. Cell Rep.

[31] Kovacs P, Schoneberg T (2016). The Relevance of Genomic Signatures at Adhesion GPCR Loci in Humans. Handb Exp Pharmacol.

[32] Ravenscroft G (2015). Mutations of GPR126 are responsible for severe arthrogryposis multiplex congenita. Am J Hum Genet.

[33] Piao X (2004). G protein-coupled receptor-dependent development of human frontal cortex. Science.

[34] Patat O (2016). Truncating Mutations in the Adhesion G Protein-Coupled Receptor G2 Gene ADGRG2 Cause an X-Linked Congenital Bilateral Absence of Vas Deferens. Am J Hum Genet.

[35] Weston MD, Luijendijk MW, Humphrey KD, Moller C, Kimberling WJ (2004). Mutations in the VLGR1 gene implicate G-protein signaling in the pathogenesis of Usher syndrome type II. Am J Hum Genet.

[36] Boyden SE (2016). Vibratory Urticaria Associated with a Missense Variant in ADGRE2. N Engl J Med.

[37] Shashidhar S (2005). GPR56 is a GPCR that is overexpressed in gliomas and functions in tumor cell adhesion. Oncogene.

[38] Tang X (2013). GPR116, an adhesion G-protein-coupled receptor, promotes breast cancer metastasis via the Galphaq-p63RhoGEF-Rho GTPase pathway. Cancer Res.

[39] Ward Y (2013). CD97 amplifies LPA receptor signaling and promotes thyroid cancer progression in a mouse model. Oncogene.

[40] Aust G, Zhu D, Van Meir EG, Xu L (2016). Adhesion GPCRs in Tumorigenesis. Handb Exp Pharmacol.

[41] Bayin NS (2016). GPR133 (ADGRD1), an adhesion G-protein-coupled receptor, is necessary for glioblastoma growth. Oncogenesis.

[42] Insel PA (2018). GPCRomics: GPCR Expression in Cancer Cells and Tumors Identifies New, Potential Biomarkers and Therapeutic Targets. Front Pharmacol.

[43] Bjarnadottir TK, Fredriksson R, Schioth HB (2007). The adhesion GPCRs: a unique family of G protein-coupled receptors with important roles in both central and peripheral tissues. Cell Mol Life Sci.

[44] Promel S (2012). The GPS motif is a molecular switch for bimodal activities of adhesion class G protein-coupled receptors. Cell Rep.

[45] Lv X (2016). *In vitro* expression and analysis of the 826 human G protein-coupled receptors. Protein Cell.

[46] Jorquera Roddy, Ortiz Rodrigo, Ossandon F., Cárdenas Juan Pablo, Sepúlveda Rene, González Carolina, Holmes David S. (2016). SinEx DB: a database for single exon coding sequences in mammalian genomes. Database.

[47] Pan Q, Shai O, Lee LJ, Frey BJ, Blencowe BJ (2008). Deep surveying of alternative splicing complexity in the human transcriptome by high-throughput sequencing. Nat Genet.

[48] Wang ET (2008). Alternative isoform regulation in human tissue transcriptomes. Nature.

[49] Bjarnadottir TK (2007). Identification of novel splice variants of Adhesion G protein-coupled receptors. Gene.

[50] Salzman GS (2016). Structural Basis for Regulation of GPR56/ADGRG1 by Its Alternatively Spliced Extracellular Domains. Neuron.

[51] Aust G, Hamann J, Schilling N, Wobus M (2003). Detection of alternatively spliced EMR2 mRNAs in colorectal tumor cell lines but rare expression of the molecule in colorectal adenocarcinomas. Virchows Arch.

[52] Stacey M, Lin HH, Hilyard KL, Gordon S, McKnight AJ (2001). Human epidermal growth factor (EGF) module-containing mucin-like hormone receptor 3 is a new member of the EGF-TM7 family that recognizes a ligand on human macrophages and activated neutrophils. J Biol Chem.

[53] Matsushita H, Lelianova VG, Ushkaryov YA (1999). The latrophilin family: multiply spliced G protein-coupled receptors with differential tissue distribution. FEBS Lett.

[54] Sugita S, Ichtchenko K, Khvotchev M, Sudhof TC (1998). alpha-Latrotoxin receptor CIRL/latrophilin 1 (CL1) defines an unusual family of ubiquitous G-protein-linked receptors. G-protein coupling not required for triggering exocytosis. J Biol Chem.

[55] Kwakkenbos MJ (2006). An unusual mode of concerted evolution of the EGF-TM7 receptor chimera EMR2. FASEB J.

[56] Boucard AA, Maxeiner S, Sudhof TC (2014). Latrophilins function as heterophilic cell-adhesion molecules by binding to teneurins: regulation by alternative splicing. J Biol Chem.

[57] Steijger T (2013). Assessment of transcript reconstruction methods for RNA-seq. Nat Methods.

[58] Pertea M, Kim D, Pertea GM, Leek JT, Salzberg SL (2016). Transcript-level expression analysis of RNA-seq experiments with HISAT, StringTie and Ballgown. Nat Protoc.

[59] Howard BE, Heber S (2010). Towards reliable isoform quantification using RNA-SEQ data. BMC Bioinformatics.

[60] Goldstein LD (2016). Prediction and Quantification of Splice Events from RNA-Seq Data. PLoS One.

[61] Trapnell C (2010). Transcript assembly and quantification by RNA-Seq reveals unannotated transcripts and isoform switching during cell differentiation. Nat Biotechnol.

[62] Dobin A, Gingeras TR (2015). Mapping RNA-seq Reads with STAR. Curr Protoc Bioinformatics.

[63] Dobin A, Gingeras TR (2016). Optimizing RNA-Seq Mapping with STAR. Methods Mol Biol.

[64] Pertea M (2015). StringTie enables improved reconstruction of a transcriptome from RNA-seq reads. Nat Biotechnol.

[65] Niknafs YS, Pandian B, Iyer HK, Chinnaiyan AM, Iyer MK (2017). TACO produces robust multisample transcriptome assemblies from RNA-seq. Nat Methods.

[66] Preissler J (2015). Altered microglial phagocytosis in GPR34-deficient mice. Glia.

[67] Engemaier E, Rompler H, Schoneberg T, Schulz A (2006). Genomic and supragenomic structure of the nucleotide-like G-protein-coupled receptor GPR34. Genomics.

[68] Schoneberg T, Meister J, Knierim AB, Schulz A (2018). The G protein-coupled receptor GPR34 - The past 20years of a grownup. Pharmacol Ther.

[69] An Dong, Cao Hieu, Li Changsheng, Humbeck Klaus, Wang Wenqin (2018). Isoform Sequencing and State-of-Art Applications for Unravelling Complexity of Plant Transcriptomes. Genes.

[70] Gunisova S, Hronova V, Mohammad MP, Hinnebusch AG, Valasek LS (2018). Please do not recycle! Translation reinitiation in microbes and higher eukaryotes. FEMS Microbiol Rev.

[71] Wang T (2005). CD97, an adhesion receptor on inflammatory cells, stimulates angiogenesis through binding integrin counterreceptors on endothelial cells. Blood.

[72] Lin HH, Stacey M, Hamann J, Gordon S, McKnight AJ (2000). Human EMR2, a novel EGF-TM7 molecule on chromosome 19p13.1, is closely related to CD97. Genomics.

[73] Gray JX (1996). CD97 is a processed, seven-transmembrane, heterodimeric receptor associated with inflammation. J Immunol.

[74] Chiang NY (2011). Disease-associated GPR56 mutations cause bilateral frontoparietal polymicrogyria via multiple mechanisms. J Biol Chem.

[75] Legrand F (2014). The eosinophil surface receptor epidermal growth factor-like module containing mucin-like hormone receptor 1 (EMR1): a novel therapeutic target for eosinophilic disorders. J Allergy Clin Immunol.

[76] Liebscher I, Schoneberg T (2016). Tethered Agonism: A Common Activation Mechanism of Adhesion GPCRs. Handb Exp Pharmacol.

[77] Bae BI (2014). Evolutionarily dynamic alternative splicing of GPR56 regulates regional cerebral cortical patterning. Science.

[78] Cardoso JC, Pinto VC, Vieira FA, Clark MS, Power DM (2006). Evolution of secretin family GPCR members in the metazoa. BMC Evol Biol.

[79] Bryson-Richardson RJ, Logan DW, Currie PD, Jackson IJ (2004). Large-scale analysis of gene structure in rhodopsin-like GPCRs: evidence for widespread loss of an ancient intron. Gene.

[80] Abe J, Suzuki H, Notoya M, Yamamoto T, Hirose S (1999). Ig-hepta, a novel member of the G protein-coupled hepta-helical receptor (GPCR) family that has immunoglobulin-like repeats in a long N-terminal extracellular domain and defines a new subfamily of GPCRs. J Biol Chem.

[81] Promel S (2012). Characterization and functional study of a cluster of four highly conserved orphan adhesion-GPCR in mouse. Dev Dyn.

[82] Tsai YS, Dominguez D, Gomez SM, Wang Z (2015). Transcriptome-wide identification and study of cancer-specific splicing events across multiple tumors. Oncotarget.

[83] Monk KR, Oshima K, Jors S, Heller S, Talbot WS (2011). Gpr126 is essential for peripheral nerve development and myelination in mammals. Development.

[84] Bridges JP (2013). Orphan G protein-coupled receptor GPR116 regulates pulmonary surfactant pool size. Am J Respir Cell Mol Biol.

[85] Brown, K. *et al*. Epithelial Gpr116 regulates pulmonary alveolar homeostasis via Gq/11 signaling. *JCI Insight* **2**, 10.1172/jci.insight.93700 (2017). 10.1172/jci.insight.93700 PMC5453702 28570277

[86] Fukuzawa T (2013). Lung surfactant levels are regulated by Ig-Hepta/GPR116 by monitoring surfactant protein D. PLoS One.

[87] Yang MY (2013). Essential regulation of lung surfactant homeostasis by the orphan G protein-coupled receptor GPR116. Cell Rep.

[88] Nie T (2012). Adipose tissue deletion of Gpr116 impairs insulin sensitivity through modulation of adipose function. FEBS Lett.

[89] Niaudet C (2015). Gpr116 Receptor Regulates Distinctive Functions in Pneumocytes and Vascular Endothelium. PLoS One.

[90] Benoit-Pilven C (2018). Complementarity of assembly-first and mapping-first approaches for alternative splicing annotation and differential analysis from RNAseq data. Sci Rep.

[91] Shao M, Kingsford C (2017). Accurate assembly of transcripts through phase-preserving graph decomposition. Nat Biotechnol.

[92] Song L, Sabunciyan S, Florea L (2016). CLASS2: accurate and efficient splice variant annotation from RNA-seq reads. Nucleic Acids Res.

[93] Krizanovic K, Echchiki A, Roux J, Sikic M (2018). Evaluation of tools for long read RNA-seq splice-aware alignment. Bioinformatics.

[94] Arac D (2012). A novel evolutionarily conserved domain of cell-adhesion GPCRs mediates autoproteolysis. EMBO J.

[95] Fredriksson R, Lagerstrom MC, Lundin LG, Schioth HB (2003). The G-protein-coupled receptors in the human genome form five main families. Phylogenetic analysis, paralogon groups, and fingerprints. Mol Pharmacol.

[96] de Graaf C, Nijmeijer S, Wolf S, Ernst OP (2016). 7TM Domain Structure of Adhesion GPCRs. Handb Exp Pharmacol.

[97] Miller RL (2015). The Importance of Ligand-Receptor Conformational Pairs in Stabilization: Spotlight on the N/OFQ G Protein-Coupled Receptor. Structure.

[98] Popov, P. *et al*. Computational design of thermostabilizing point mutations for G protein-coupled receptors. *Elife* **7**, 10.7554/eLife.34729 (2018). 10.7554/eLife.34729 PMC6013254 29927385

[99] Chun E (2012). Fusion partner toolchest for the stabilization and crystallization of G protein-coupled receptors. Structure.

[100] Kodama Y, Shumway M, Leinonen R (2012). & International Nucleotide Sequence Database, C. The Sequence Read Archive: explosive growth of sequencing data. Nucleic Acids Res.

[101] Li H (2009). The Sequence Alignment/Map format and SAMtools. Bioinformatics.

[102] Robinson JT (2011). Integrative genomics viewer. Nat Biotechnol.

[103] Thorvaldsdottir H, Robinson JT, Mesirov JP (2013). Integrative Genomics Viewer (IGV): high-performance genomics data visualization and exploration. Brief Bioinform.

[104] Breese MR, Liu Y (2013). NGSUtils: a software suite for analyzing and manipulating next-generation sequencing datasets. Bioinformatics.

[105] Quinlan AR, Hall IM (2010). BEDTools: a flexible suite of utilities for comparing genomic features. Bioinformatics.

[106] Quinlan AR (2014). BEDTools: The Swiss-Army Tool for Genome Feature Analysis. Curr Protoc Bioinformatics.

[107] Charif, D. & Lobry, J. R. In *Structural approaches to sequence evolution: Molecules, networks, populations Biological and Medical Physics, Biomedical Engineering* (eds Bastolla, U., Porto, M., Roman, H. E. & Vendruscolo, M.) 207–232 (Springer Verlag, 2007).

[108] Apweiler R (2004). UniProt: the Universal Protein knowledgebase. Nucleic Acids Res.

[109] Team, R. D. C. R*:**A Language and Environment for Statistical Computing*, http://www.r-project.org/ (2008).

[110] Hoffmann S (2009). Fast mapping of short sequences with mismatches, insertions and deletions using index structures. PLoS Comput Biol.

[111] Hoffmann S (2014). A multi-split mapping algorithm for circular RNA, splicing, trans-splicing and fusion detection. Genome Biol.

[112] Otto C, Stadler PF, Hoffmann S (2014). Lacking alignments? The next-generation sequencing mapper segemehl revisited. Bioinformatics.

[113] Demberg LM (2017). Activation of Adhesion G Protein-coupled Receptors: Agonist specificity of stachel sequence-derived peptides. J Biol Chem.

[114] Schoneberg, T. *et al*. V2 vasopressin receptor dysfunction in nephrogenic diabetes insipidus caused by different molecular mechanisms. *Hum Mutat* **12**, 196-205, doi:10.1002/(SICI)1098-1004(1998)12:3<196::AID-HUMU7>3.0.CO;2-F (1998). 10.1002/(SICI)1098-1004(1998)12:3<196::AID-HUMU7>3.0.CO;2-F 9711877

[115] Halvardson J, Zaghlool A, Feuk L (2013). Exome RNA sequencing reveals rare and novel alternative transcripts. Nucleic Acids Res.

[116] Jones DT, Taylor WR, Thornton JM (1992). The rapid generation of mutation data matrices from protein sequences. Comput Appl Biosci.

[117] Kumar S, Stecher G, Tamura K (2016). MEGA7: Molecular Evolutionary Genetics Analysis Version 7.0 for Bigger Datasets. Mol Biol Evol.

