# Supplementary material for: Genetic basis of functional variability in adhesion G protein-coupled receptors
Source: Sci Rep. 2019 Jul 30;9:11036. doi: 10.1038/s41598-019-46265-x (PMC6667449; doi:10.1038/s41598-019-46265-x)
Supplement: Supplementary file 1 — Supplementary [file 41598_2019_46265_MOESM1_ESM.pdf]

Supplementary Information

# Genetic basis of functional variability in adhesion G protein-coupled receptors

Alexander Bernd Knierim[1,2], Juliane Röthe[1,2], Mehmet Volkan Çakir[1], Vera Lede[1], Caroline Wilde[1], Ines Liebscher[1], Doreen Thor[1,2], and Torsten Schöneberg[1]

[1]Rudolf Schönheimer Institute of Biochemistry, Molecular Biochemistry, Medical Faculty, University of Leipzig, 04103 Leipzig, Germany.
[2]Leipzig University Medical Center, IFB AdiposityDiseases

## Results

### *Further validation of the variant detection pipeline*

To determine the FPKM value that reflects significant expression above noise we plotted the average FPKM value of each mouse gene and determined the median FPKM value of all genes (FPKM = 0.42) (Figure S2). All genes showing an FPKM ≥0.5 were considered as significantly expressed above noise.

To extract a maximum of full-length mRNA variants a certain number of non-redundant reads covering the gene locus is required. To estimate the number of ADGRF5/GPR116 locus-specific reads necessary to yield a saturated variant analysis we randomly extracted permutated subsets of reads from the RNA-seq libraries (0-300 million reads) and plotted the read number against the number of variants which were extracted from the subsets by our *de novo* assembly pipeline. Theoretically, we expected a V-shaped curve (suppl. Figure S3A), where, at a low number of reads, only fragments of full-length transcripts will appear implying a high number of variants (false positive). Increasing the number of reads will first reduce the number of variants because fragments are assembled together by exon-exon read support but full-length assembled variants are rather rare in such library subsets. Further increasing the read number will lead to longer and finally full-length variants until saturation. The curve of the *de novo* assembly of transcript variants depending on the read number used for the analysis exactly shows the V-shaped dependency (suppl. Figure S3B). When using about 100 million reads of the whole RNA-seq library (refers to approximately 41,000 ADGRF5/GPR116 locus-specific reads) saturation of the number of splice variants is found. This indicates that for ADGRF5/GPR116 the RNA-seq libraries from visceral adipose tissue (VAT) are suitable for a saturating splice variant analysis.

We next analyzed the islet RNA-seq dataset where ADGRF5/GPR116 expression was close to the cut-off FPKM ≥0.5. As shown in suppl. Figure S3B (blue curve), random extraction of different read numbers and analysis revealed again a V-shaped curve. Inspection of the *de novo* assembled transcripts revealed a very similar transcript length distribution as seen for the analysis of ADGRF5/GPR116 in VAT (suppl. Table S3).

An FPKM value above 0.5 does not always guarantee for a proper analysis. For example, AdgrE4/Emr4 had an FPKM >0.5 in visceral adipose tissue (VAT) but we did not observe saturation in the predicted variant number in this analysis. Therefore, we excluded this dataset. The same was true for aGPCRs close to the FPKM cutoff (e.g. Adgrl3/Lphn3 in VAT, Adgrd1/Gpr133 in liver and Adgrg6/Gpr126 in islets, see Table S2).

To finally assure that the number of variants is not a function of the FPKM we performed the pipeline for all 18 aGPCRs with an FPKM value ≥0.5 at least in one of the three tissues. As

shown in suppl. Figure S4 there is no correlation between the number of aGPCR variants and the respective FPKM. Even for the individual aGPCR there was no correlation of the variant number in different tissues and the respective FPKM (see suppl. Tables S2 and S3).

### *Evolutionary conservation of alternative splice events*

Because evolutionary conservation of splice variants may provide further support to the validity of found variants, we exemplarily asked whether mouse ADGRF5/GPR116 transcripts are species-specific or also present in human and other species. For example, the variable exon 79 (see Figure 1 and Figure 2: Δ between Ig domains 2 and 3) is also found in human ADGRF5/GPR116 transcripts (XP_016865910.1 vs. XP_016865911.1). Similarly, sequence length variability of the C terminus is also found in human ADGRF5/GPR116 variants (compare XP_016865910.1, XP_016865911.1, XP_016865913.1). The same variability in the N- and C termini of ADGRF5/GPR116 is, for example, found in rat (XP_008765055.1, XP_008765056.1 XP_008765056.1) and cow (XP_005223514.1, XP_005223510.1, XP_005223514). Besides these common alternative exon usages, we also found more rare variants by reevaluation of transcripts in NCBI. In mouse for example, usage of exon 81 (see Figure 1) causes premature truncation of the N terminus (ADGRF5-8/-16) or late start encoding for a CTF (ADGRF5-7). The homologous exon is also used in human ADGRF5/GPR116 (e.g. AK300380) and rat (CK473917.1) transcripts but not annotated as an isoform, yet. It, therefore, appears that many ADGRF5/GPR116 splice variants are evolutionarily conserved in mammals supporting their physiological significance.

Supplementary Figures

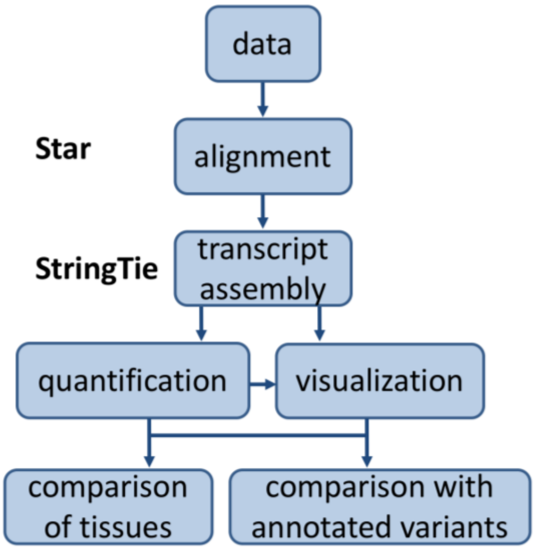


**Suppl. Figure S1 General pipeline to extract aGPCR transcript variants from Illumina RNA-seq data.**

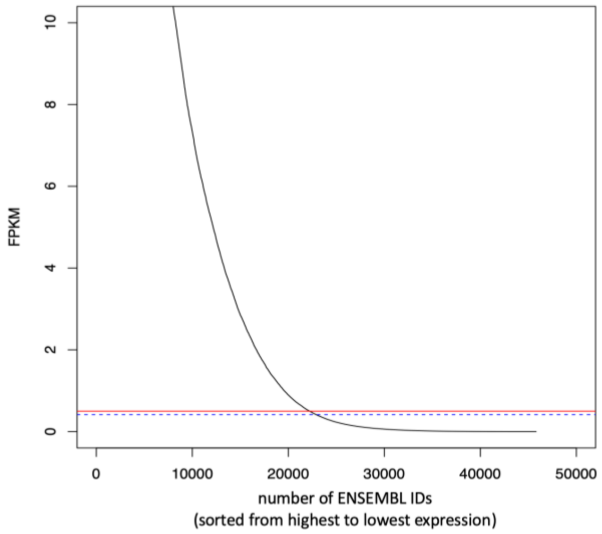


**Suppl. Figure S2 Determination of the FPKM cut-off.**
All annotated mouse ENSEMBL IDs (x axis) ranked by their average expression in all tissues was plotted against their respective mean FPKM. Note, the number of mouse genes is lower than the number of ENSEMBL IDs because many genes have more than one ENSEMBL ID and several transcripts are not assigned to a specific gene. The blue dotted line is the median FPKM value of all genes (0.42). For simplicity, we used a FPKM value of ≥0.5 as an inclusion criteria for analysis (red line).

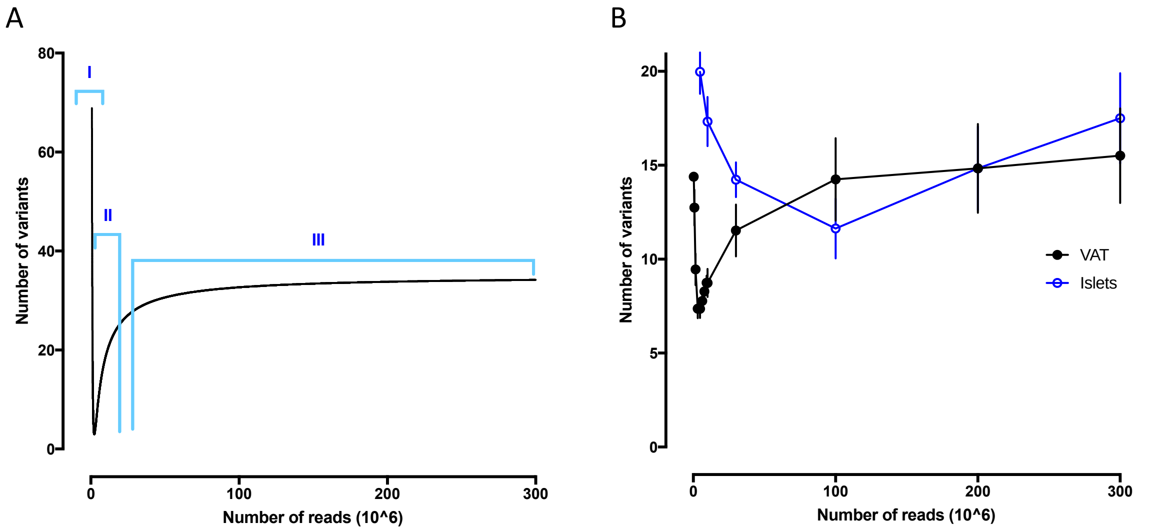


**Suppl. Figure S3 Theoretical (A) and experimentally determined (B) curves of the number of splice variants depending on the number of RNA-seq reads.**

**A**) The curve shown represents the theoretical expectation from *de novo* assembly of splice variants from fragmented mRNA sequences. Assembly of splice variants from RNA-seq reads (x axis) requires a certain number of gene-specific reads to generate a full-length transcript. Therefore, starting from >0 the number of "variants" reflects the gene-specific reads that are unique and do not assemble to full-length transcripts representing just the number of transcript fragments (part I of the curve). Increasing numbers of gene-specific reads will assemble to longer transcripts and then the first complete full-length transcripts reduce the variant quantity (part II of the curve). High read coverage of a gene will lead to an increasing number of full-length variants reaching saturation (part III of the curve). **B**) Reads (0.3, 1, 3, 5, 6, 8, 9, 10, 30, 100, 200, 300 million reads) were randomly taken from the original RNA-seq dataset (up to 100 permutations) (ADGRF5/GPR116: visceral adipose tissue (VAT), pancreatic islet) and receptor variants were extracted using the annotation pipeline described above. The aGPCR variant number is plotted against the read number of the randomly chosen sub-libraries. FPKM values ± SD: 50.8 ± 9.1 (VAT, n=3) and 4.9 ± 0.1 (islet, n=3) (suppl. Table S2).

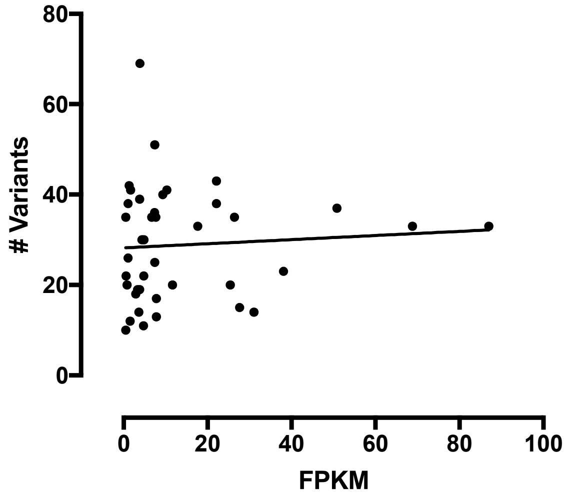


**Suppl. Figure S4 Analysis of the relation between FPKM and the number of mRNA variants.**

Data from the mRNA variant analysis pipeline of 18 aGPCR (see data sheet of the individual genes) and the respective FPKM value in the analyzed tissue (suppl. Table S2) are plotted. There is no significant correlation between FPKM (Pearson correlation coefficient r=0.068) and the number of variants using the described inclusion parameters.

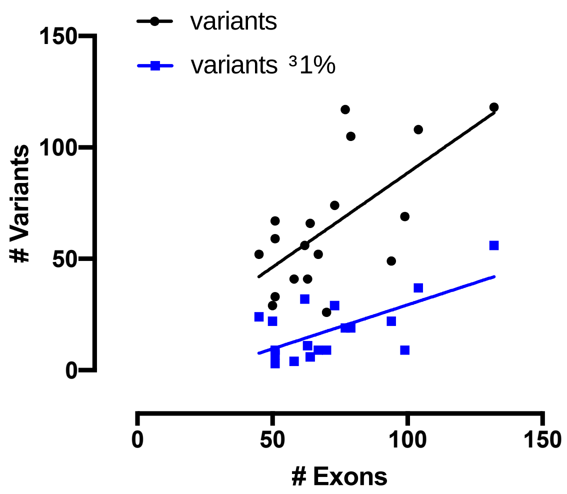


**Suppl. Figure S5 Analysis of the relation between the number of exons and the number of mRNA variants.** The number of mRNA variants taken from the analysis pipeline of 18 aGPCRs and the respective exon number (suppl. Table S4) of the individual genes are plotted. There is a positive correlation between the number of exons (Pearson correlation coefficients: r=0.66 all variants, r=0.65 variants with an abundance of ≥1% of all specific aGPCR transcript variants) and the number of variants.

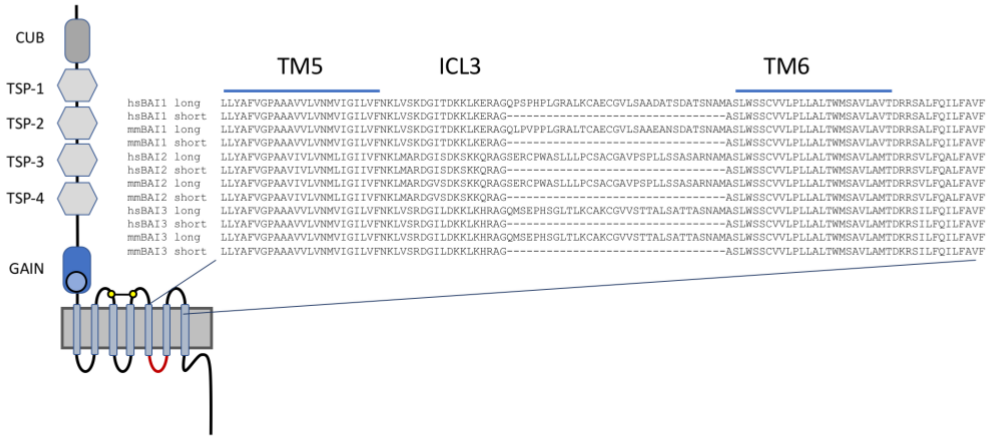


**Suppl. Figure S6 Loop length variations in members of the ADGRB group.** All members of the ADGRB group have splice variants conserved between mouse and human changing the length of the 3. intracellular loop. The amino acid sequence alignment of the region between transmembrane helix 5 and 6 (TM5, TM6) of the human (*Homo sapiens*, hs) and mouse (*Mus musculus*, mm) BAI1-3 orthologs is shown. CUB, complement C1r/C1s, Uegf, Bmp1 domain; GAIN, G protein-coupled receptor autoproteolysis-inducing domain; ICL3, 3. intracellular loop; TM, transmembrane helix; TSP, thrombospondin domain

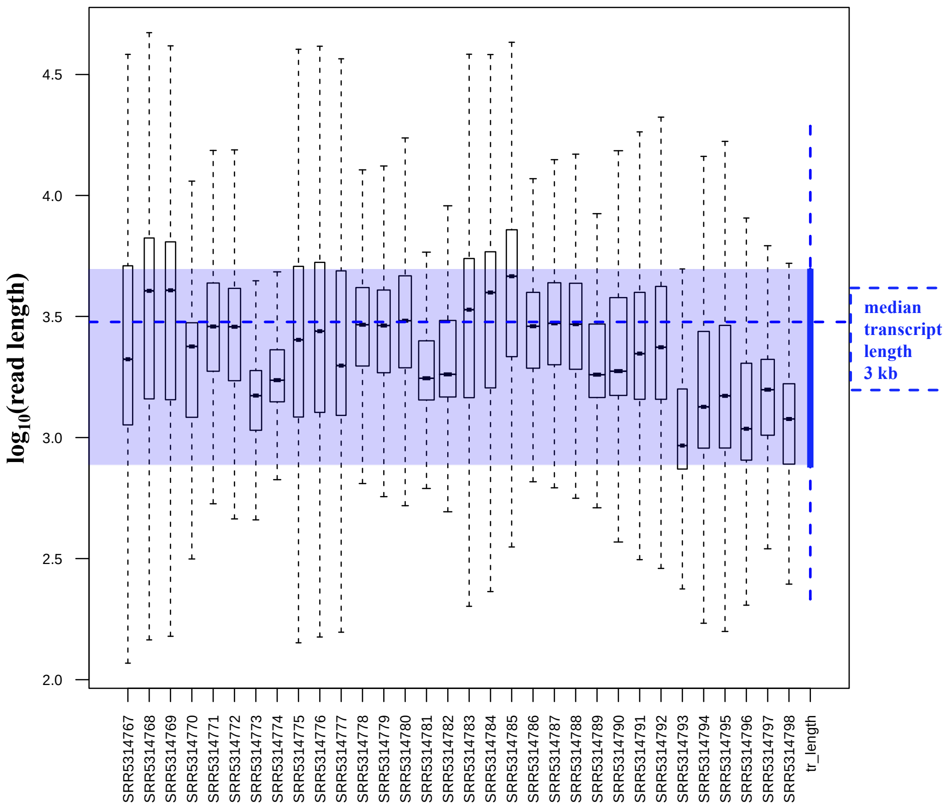


**Suppl. Figure S7 Clipped read length distribution of PacBio samples.** It is shown that distribution of read lengths covers the transcript-length distribution nicely. Transcript length distribution was calculated by summing the length of exons for each transcript variant for the genes of interest.


1 Steijger, T. *et al.* Assessment of transcript reconstruction methods for RNA-seq. *Nat Methods* **10**, 1177-1184, doi:10.1038/nmeth.2714 (2013).

2 Goldstein, L. D. *et al.* Prediction and Quantification of Splice Events from RNA-Seq Data. *PLoS One* **11**, e0156132, doi:10.1371/journal.pone.0156132 (2016).

3 Pertea, M., Kim, D., Pertea, G. M., Leek, J. T. & Salzberg, S. L. Transcript-level expression analysis of RNA-seq experiments with HISAT, StringTie and Ballgown. *Nat Protoc* **11**, 1650-1667, doi:10.1038/nprot.2016.095 (2016).

4 Howard, B. E. & Heber, S. Towards reliable isoform quantification using RNA-SEQ data. *BMC Bioinformatics* **11 Suppl 3**, S6, doi:10.1186/1471-2105-11-S3-S6 (2010).

5 Trapnell, C. *et al.* Transcript assembly and quantification by RNA-Seq reveals unannotated transcripts and isoform switching during cell differentiation. *Nat Biotechnol* **28**, 511-515, doi:10.1038/nbt.1621 (2010).

# Supplementary Tables

**Table S1**
***Parameters of RNA-seq datasets used in this study.***
The GEO accession number, the BioProject identification number, the number of raw reads, and the number of uniquely aligned reads are given.

**Table S2**
***FPKM values of aGPCR in tissues.***
The determined FPKM values of the individual aGPCRs in each samples of visceral adipose tissue (VAT), liver, and pancreatic islets together with the means ± SD are given. Samples <0.5 FPKM and/or absence of long transcript assemblies (grey boxes) are excluded from the analyses.

**Table S3**
***Variant quantification and exon usage of individual aGPCR analyzed in this study.***
The identified exons (numbering referred to the reference mouse genome (mm10/GRCm38)), the exon composition of transcripts, the transcript abundance, the already annotated exons and transcripts (accession number) are given for each of the 18 analyzed aGPCR genes.

**Table S4**
***Overall variant quantification and exon usage in aGPCR splice variants***
This table is an extension of data given in Table 2. Already known and newly identified exons are listed and analyzed. 5' start exons with minor differences in the transcription start site but identical 3' splice donor sites are considered as one 5' start exon. Similarly, 3' end exons with minor differences in length but identical 5' splice acceptor sites are considered as one 3' end exon. Different composition of the 5' start exon, 3' end exon and/or exons are considered as individual variants. Exons are defined by a donor site and an acceptor site.
